# Complexity of a small non-protein coding sequence in chromosomal region 22q11.2: presence of specialized DNA secondary structures and RNA exon/intron motifs

**DOI:** 10.1186/s12864-015-1958-6

**Published:** 2015-10-14

**Authors:** Nicholas Delihas

**Affiliations:** Department of Molecular Genetics and Microbiology, School of Medicine, Stony, Brook University, Stony Brook, NY 11794 USA

**Keywords:** Chromosome region 22q.11.2, Translocation breakpoint sequences, Biased mutations, DNA secondary structure, RNA exons, Introns

## Abstract

**Background:**

DiGeorge Syndrome is a genetic abnormality involving ~3 Mb deletion in human chromosome 22, termed 22q.11.2. To better understand the non-coding regions of 22q.11.2, a small 10,000 bp non-protein-coding sequence close to the DiGeorge Critical Region 6 gene (*DGCR6*) was chosen for analysis and functional entities as the homologous sequence in the chimpanzee genome could be aligned and used for comparisons.

**Methods:**

The GenBank database provided genomic sequences. In silico computer programs were used to find homologous DNA sequences in human and chimpanzee genomes, generate random sequences, determine DNA sequence alignments, sequence comparisons and nucleotide repeat copies, and to predicted DNA secondary structures.

**Results:**

At its 5′ half, the 10,000 bp sequence has three distinct sections that represent phylogenetically variable sequences. These Variable Regions contain biased mutations with a very high A + T content, multiple copies of the motif TATAATATA and sequences that fold into long A:T-base-paired stem loops. The 3′ half of the 10,000 bp unit, highly conserved between human and chimpanzee, has sequences representing exons of lncRNA genes and segments of introns of protein genes. Central to the 10,000 bp unit are the multiple copies of a sequence that originates from the flanking 5′ end of the translocation breakpoint Type A sequence. This breakpoint flanking sequence carries the exon and intron motifs. The breakpoint Type A sequence seems to be a major player in the proliferation of these RNA motifs, as well as the proliferation of Variable Regions in the 10,000 bp segment and other regions within 22q.11.2.

**Conclusions:**

The data indicate that a non-coding region of the chromosome may be reserved for highly biased mutations that lead to formation of specialized sequences and DNA secondary structures. On the other hand, the highly conserved nucleotide sequence of the non-coding region may form storage sites for RNA motifs.

**Electronic supplementary material:**

The online version of this article (doi:10.1186/s12864-015-1958-6) contains supplementary material, which is available to authorized users.

## Background

DiGeorge Syndrome is part of a group of genetic disorders that occur in humans termed 22q11.2 deletion syndrome [1]. The DiGeorge disorder involves ~3 Mb deletion of the chromosomal 22q11.2 region that results in loss of approximately ~40 protein genes. Clinical manifestations of a 22q11.2 deletion are pleiotropic and can include congenital heart disease, developmental problems, neurological abnormalities, immune system abnormalities, learning disabilities and psychiatric problems that also includes autism [1–4].

Deletions in chromosomal region 22q11.2 can occur from an unequal crossover between low copy repeats (LCR)s present in this region of chromosome 22. LCRs can contain very long A + T-rich stem loops that are termed palindromic A + T-rich repeats (PATRR) [5–11]. These have been cloned and sequenced and appear to be central to translocation [12]. PATRRs can form cruciforms and are prone to breakage that can lead to chromosomal translocation. The stem loop sequences are also referred to as translocation breakpoint hot spots. Some of these have flanking sequences that are conserved or partially conserved between closely related PATRR elements.

To better understand the nature of non-coding genomic regions in a 22q.11.2 deletion, an analysis was made of a 10,000 bp non-protein coding region (GRCh38 primary assembly, coordinates:18890337–18900336), which is just upstream of the DiGeorge Critical Region 6 gene (*DGCR6*). *DGCR6* is homologous to a *Drosophila melanogaster* gene, whose protein product may participate in gonadal cell development and other cell functions [13]. The 10,000 bp region, albeit representing a small fraction of the 3 Mb region, was chosen for analysis as the homologous chimpanzee sequence for the most part has been determined. Importantly, the *DGCR6* gene has been annotated in both species and this provided the guide post for alignment of sequences between species. In addition, alignment was possible even with the presence of redundant sequences (low-complexity sequences), and thus, comparisons that reveal high sequence identity and/or variation could be made. Central to this region and other regions of 22q11.2 is the presence of multiple copies of segments of the Translocation Breakpoint Type A sequence (GenBank, AB261997) [12]. We find numerous segments of translocation breakpoint flanking sequence insertions within the 10,000 bp region, but these lack the breakpoint hot spot stem loop sequences (PATRRs). Several of the breakpoint flanking sequences are not present in the homologous chimpanzee region. This enabled us to delineate three sequence Variable Regions, #1-#3 and these are in the 5′ half of the 10,000 bp unit.

The three phylogenetically Variable Regions are very A + T-rich, in both humans and chimpanzee. These regions mutate very rapidly, however, in humans, mutations significantly accelerate the formation of a nine bp sequence, TATAATATA [9] and there also are sequences that fold into long A:T-rich stem loops, which are better formed and longer in humans relative to the chimpanzee. Of importance, the 22q11.2 region is one of the most susceptible regions of the human genome to undergo genetic recombination [12], although the 10,000 bp region is not in the region observed to undergo repeated translocation, which is the LCR-B chromosomal region [10].

To add to the complexity of the 10,000 bp region, we find that translocation breakpoint flanking sequences are carriers of a specific exon sequence that is present in long non-coding RNAs (lncRNA)s and in addition, segments of mRNA intron sequences; these are abundantly found in the 10,000 bp unit. The motifs may represent reservoirs for use in possible formation of new lncRNA and protein genes.

This paper presents new and multifaceted concepts in genomics, which include the spread of RNA exon and intron motifs within the genome by duplication of translocation breakpoint sequences, genomic regions reserved for translocation breakpoint sequences that may participate in formation of highly biased mutations in A + T-rich regions, and phylogenetically stable storage regions in the genome that contain both lncRNA exon and mRNA intron sequences.

## Results

**Fig. 1:**
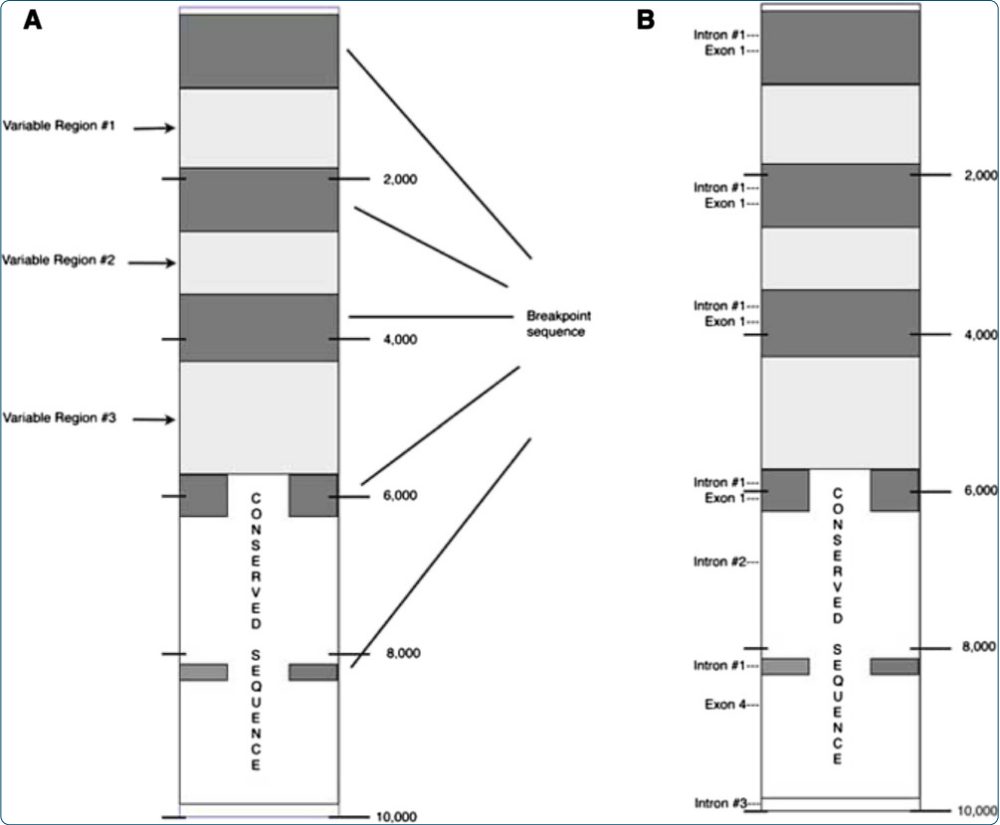
Schematic of the human 10,000 bp region uptream of *DGCR6.* **a** Locations of breakpoint flanking sequences. **b** Locations of exon 1 and exon 4 and introns #1-#3

The 3′ end of the 10,000 bp non-coding region is situated 6039 bp upstream of the *DGCR6* gene in Homo sapiens chromosome 22 (GRCh38 Primary Assembly, coordinates:18890337–18900336). This region has blocks of repeats and redundant sequences that are of low complexity and these create problems in sequence alignment. However, alignment in the 10,000 bp region was possible because of the presence of translocation breakpoint sequence inserts that serve as guideposts. Alignment of sequences with the analogous chimpanzee sequence, which is 5892 bp upstream of the *DGCR6* isoform 1 gene (Pan troglodytes chromosome 22, Pan_troglodytes-2.1.4, coordinates: 17300774 to 17307562 ) is shown in Additional file 1: Figure S1, and a schematic of the 10,000 bp region is in Fig. 1. Three global alignment programs [14–16] were employed to verify the accuracy of the overall alignment. “Edge effects” at A + T-rich redundant regions occur with different alignment programs but this had a negligible effect on the overall alignment pattern as the translocation breakpoint flanking sequence inserts, which are present in the human sequence but are mostly missing in the chimpanzee region (see Additional file 1: Figure S1), sufficiently delineate the variable sequence regions, serve as guideposts and allow for Variable Region analyses.

Alignment at the 5′ half of the human 10,000 bp segment shows three significant variable sequence blocks, termed Variable Regions #1- #3 (Fig. 1a). The inserts are breakpoint Type A flanking sequences, and these are a major component of the 5′ half of the human 10,000 bp unit. However, the nucleotide sequence lengths within each Variable Region differ when the human sequence is compared with the homologous chimpanzee sequence. This is not due to breakpoint sequence additions, but due to other added sequences that are only present in the human segments of the Variable Regions. There are no additions in the comparable chimpanzee regions, e.g., see human positions 911–1953, Additional file 1: Figure S1.

On the other hand, the 3′ half of the 10,000 segment shows a very high nucleotide sequence identity (97 %) between human and chimpanzee sequences, however, there are two partial breakpoint flanking sequences present in both the human and chimpanzee regions and they are also conserved phylogenetically. Figure 1b shows the locations of intron and exon sequences that are present in the 10,000 bp unit.

### Translocation breakpoint sequences and secondary structures

Collaborations between Japanese and American investigators resulted in pioneer work on the characterization of translocation breakpoint hot spot secondary structures and functions of these structures in genetic exchange [10, 17, 18]. In addition, another group, by using biophysical calculations has shown that translocation frequency is very closely related to stem loop ability to form DNA cruciform structures [19]. One palindromic sequence found on chromosomal segment 22q11.2 is Type A (NCBI GenBank: AB261997.1). Two variations, Types B and C are also known. They have minor sequence changes, however Type A has a repeat of the first 363 bp of the 5′ end sequence at its 3′ end sequence. In addition, breakpoint sequences present on chromosome 11 have also been well-characterized [7]. PATRR breakpoint hot spot sequences fold into very long stem-loops [7, 10, 20, 21]. A total of twelve PATRR sequences and their translocation frequencies have been described [10]. We analyzed secondary structures of the twelve PATRR sequences, however, two PATRRs that exhibit extreme examples of translocation frequency are described here. These two differ by over a factor of 100 in translocation frequency [10]. The Chr 22 TYPE C (accession number AB538237.2) sequence shows a near perfect 294 bp stem, two TATAATATA motifs on the stem situated close to the top apex loop, but has an internal bulge with 3 nt on both sides of the stem (Additional file 2: Figure S2). The PATRR structure TYPE C from Chr 22 exhibits one of the highest translocation frequencies [10]. In contrast, a PATRR sequence from Chr 11 (accession number AF391128) is one of four PATRRS that exhibits a very low frequency of translocation [10]. Its predicted secondary structure shows a more imperfect stem with two large looped out regions, has a much smaller stem (87 bp), but it does have a TATAATATA motif close to the top apex loop on the 3′ side (Additional file 3: Figure S3).

A comparison of secondary structures and translocation frequencies of the twelve PATRR sequences suggests the following for PATRRS that display a high frequency of translocation. A near perfect long stem consisting of ~200 bp or more, a small top apex loop (<5 nt), greater than 90 % A:T base pairs in the upper third of the stem and a moderate abundance (~40 %) of G:C pairs in the bottom 2/3 of the stem appear to be important. Small internal loops in the stem are tolerated, but the presence of large internal loops, protruding stem loops, or short stems do not appear be to conducive to high frequency translocation. The TATAATATA sequence close to the top apex loop is common to most PATRRs. However, the motif is not found in all PATRR structures that exhibit translocation, e.g. the PATRR stem loop in NCBI Gene Bank accession #AB235190 nt sequences, albeit this example displays a low frequency of translocation [7].

The translocation breakpoint Type A, in addition to carrying the A + T palindromic breakpoint sequence, surprisingly contains two unrelated motifs; these reside on the 5′ flanking side of the breakpoint hot spot sequence (Additional file 4: Figure S4). These are RNA motifs that consist of an exon sequence found as exon 1 in different lncRNA transcripts with a high sequence identity, and a partial sequence of an intron found in different mRNA transcripts, also with a high identity.

### 10,000 bp Unit Variable Regions

An analysis was made of the three Variable Regions found in the 5′ half of the human 10,000 bp unit that are very rich in A + T residues. In addition, the number of copies of the 9 nt sequence TATAATATA [12] was determined in all three Variable Regions. Results show that there are multiple copies of the TATAATATA motif present in each of the three Variable Regions, but humans contain a significantly larger number than present in the chimpanzee (Table 1). There appears to be a marked bias towards adding and/or maintaining the TATAATATA motif in Variable Regions in both human and chimpanzee genomes. For example, human Region #1 contains 94 % A + T residues with a length of 1054 nt. Twenty-five random sequence samples with the same parameters (A + T percentage and length) show that on the average, 0.8 copies of TATAATATA/random sample, whereas Variable Region #1 in humans has 38 and the chimpanzee has 20 (Table 1). The p-value for the human TATAATATA copy number (38) vs the copy number from random sequences (0.8) has been determined by a conservative nonparametric test, Wilcoxon’s signed rank test [22]. The p-value is <0.0001. Thus, the human sequence has an almost a 50-fold greater number of the conserved 9 bp sequence relative the 25 random sequence samples. However, the Variable Regions also maintain a high copy number of a few very closely related sequences such as TATTATATA as well (data not shown); thus, a bias extends to closely related sequences as well.

**Table 1:** TATAATATA copies in DNA variable regions

Variable Region	Human coordinates	TATAATATA copies		Ratio^a^
		Human chimpanzee		
1.	911-1964	38	20	1.9
2.	2753-3484	20	8	2.5
3.	4365-5760	26	23	1.1

^a^Ratio of TATAATATA copies, human/chimpanzee

**Fig. 2:**
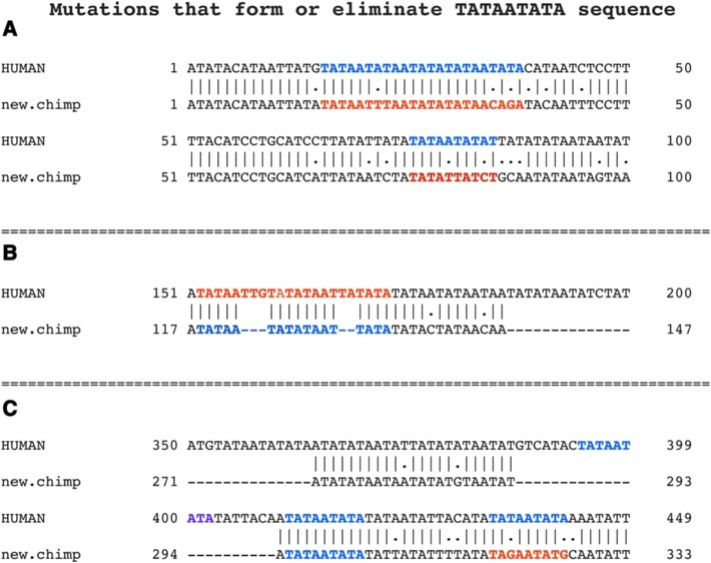
Alignment of human and chimpanzee sequences that shows formation or loss of TATAATATA motif. Three separate alignment programs gave similar alignments in the small genomic segments shown above. The nature of mutations, whether a base change, sequence addition or oligo-insertion in the human sequence occurred is shown. Color code: blue, TATAATATA motif formed, red, mutations destroy motif. **a** bp substitution creates TATAATATA motif in human sequence (however, we can not rule out a point mutation in the chimpanzee sequence destroyed a TATAATATA motif). **b** Oligo-bp insertions in human sequence eliminates two overlapping TATAATATA motifs. **c** Oligo-bp insertion in human sequence adds motif, another motif is conserved between two species, and two bp substitutions create a motif in human. Alignments are by Emboss Needle (www.ebi.ac.uk/Tools/psa/emboss_needle/)

A comparison of aligned sequences in the Variable Regions shows that the human sequence has expanded greatly compared to the chimpanzee; however, the additional sequences are not related to translocation breakpoint flanking sequences. Analyses of alignments show that both point mutations and the additional sequences in the human genome contribute to the greater number of TATAATATA copies in the human sequence. Figure 2 shows examples of bp mutations and/or addition of nt sequences that create as well as destroy the TATAATATA motif in human sequences compared to that of the chimpanzee, as well as a conservation of the motif between the two species.

**Fig. 3:**
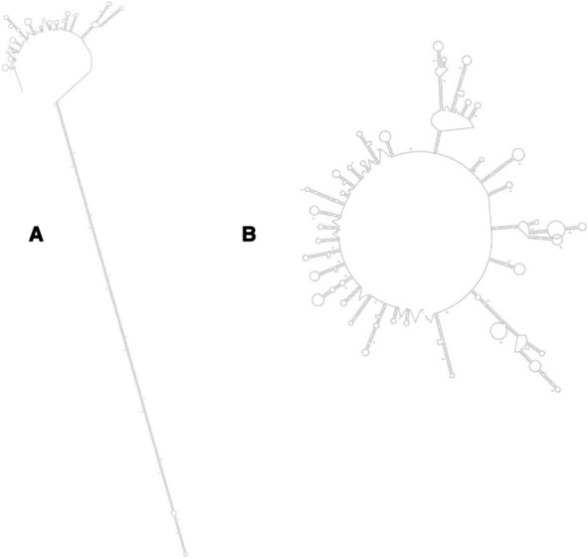
Predicted DNA secondary structures generated by mfold (http://mfold.rna.albany.edu/?q=mfold/dna-folding-form). **a** Type C PATRR. Sequence from Accession:AB538237.2. **b** Random sequence determined by use of Molbiol.ru: (http://molbiol.ru/eng/scripts/01_16.html) and generated from 1393 nucleotides with 8.3 % G + C

**Fig. 4:**
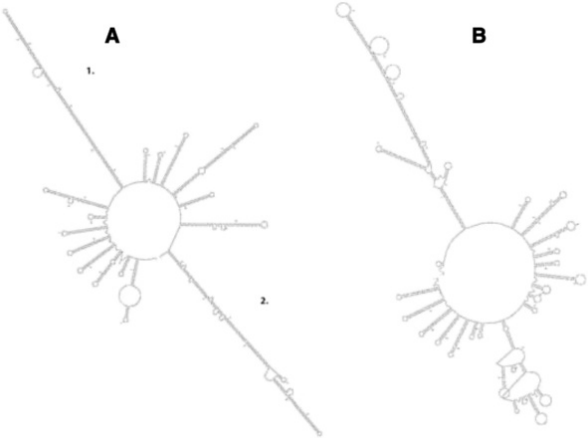
Predicted DNA secondary structures generated by mfold (http://mfold.rna.albany.edu/?q=mfold/dna-folding-form). **a** Sequence from human Variable Region #3; **b** Sequence from chimpanzee Variable Region #3

Variable regions were also analyzed for DNA secondary structure features. The structure of Chr22 TYPE C Accession:AB538237.2 PATRR (Fig. 3a), which displays a very high frequency of translocation [10] is used here as a model for DNA secondary structure and high translocation. In addition, the predicted structure for a typical sample random sequence is also shown (Fig. 3b). All three Variable Regions of the human 10,000 bp segment show at least one long A:T base pair-rich stem loop structure, albeit the human Variable Region #1 sequence folds into a poorly formed stem loop. We use the sequence from Variable Region #3 as a model, which displays the best-formed stem loops. Human and chimpanzee predicted secondary structures from this region are shown in Fig. 4a and B, respectively. The human structure shows two long stem loops that are fairly well formed; the chimpanzee has one. The lengths of the human stem loops are 120 bp (stem loop 2) and 109 bp (stem loop 1); the chimpanzee structure shows 106 bp (Table 2). A comparison of the long stem loops between human and chimpanzee shows that the human structures have fewer looped out regions and smaller stem protruding “mini-stem loops” (Fig. 4). The entire Variable Region #3 is also more thermodynamically stable in humans than in chimpanzee, 257 kcal/mol vs 164 kcal/mol, respectively (Table 3) but the Gibbs free energies for the stem loops alone are only moderately more stable in human samples compared to the chimpanzee. Overall, the human stem loop structures are much closer to the model translocation hot spot structure (Fig. 3a) than that of the chimpanzee.

**Table 2:** Properties of stem loops Variable Region #3

Species/Sequence	stem loop	Base pairs	TATAATATA near loop	Delta G
Human	1	109	No	−51 kcal/mol
	2	120	Yes	−52 kcal/mol
Chimpanzee	1	106	Yes	−44 kcal/mol.
Breakpoint				
Type C	1	294	Yes	−319 kcal/mol
Random				
Sequence	1^a^	123	No	−19 kcal/mol

^a^Stem loop shown in Additional file 6: Figure S5

**Table 3:** Total Variable #3 Region

Human	−257 kcal/mol
Chimpanzee	−164 kcal/mol
Breakpoint Type C	−374 kca,/mol
Random Sequence^a^	−120 kcal/mol

^a^From Fig. 4b

**Fig. 5:**
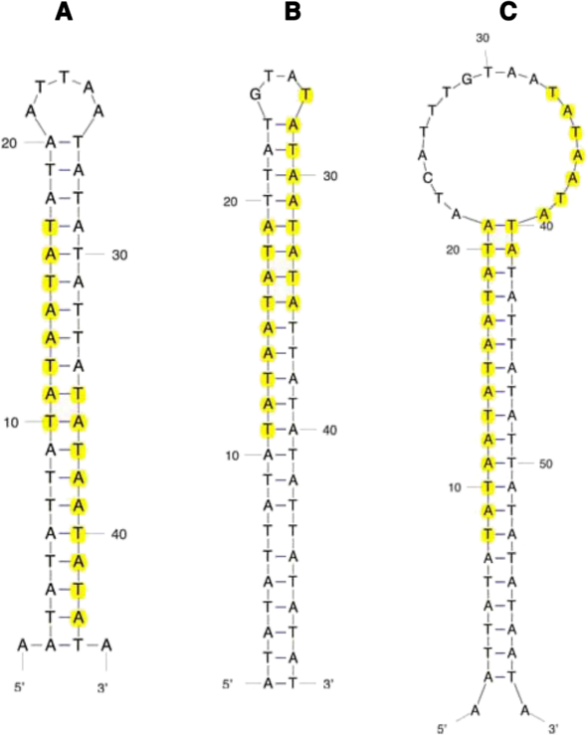
Predicted apex structures of stem loops determined by mfold (http://mfold.rna.albany.edu/?q=mfold/dna-folding-form). Yellow highlighted bases signify the conserved TATAATATA motif. **a** Apex stem loop from Fig. 3a Type C PATRR, NCBI Accession number AB538237.2. **b** Apex stem loop from human stem loop 2 of Fig. 4a. **c** Apex stem loop from chimpanzee stem loop of Fig. 4b

The apex “hairpin loop” structure (Fig. 5) is also better formed in humans compared to that of the chimpanzee with 4 bases comprising the single stranded loop for the human loop but 18 for the chimpanzee loop (Fig. 5b, c), but both human (stem loop 2) and the chimpanzee have TATAATATA motifs on the 5′ and 3′ sides of the loop, and the chimpanzee stem loop has 3 copies, two of which overlap on the 5′ side. The model translocation breakpoint Type C stem loop has 5 bases in the apex loop and TATAATATA motifs on both 5′ and 3′ sides of the loop (Fig. 5a)

A striking difference between Variable Region #3 stem loops and the breakpoint Type C stem loop is the sharp contrast in stem loop delta G values (Table 2). This is due to the greater number of bp and a much greater number of G:C pairs present in the Type C hairpin stem. Translocation Type C stem has ~5 times as many G:C pairs/bp as human stem loop 2 (Table 4). The chimpanzee stem has about a tenth of Type C stem. G:C pairs may be crucial to maintaining the stem, as its upper apex portion may extensively breathe or unfold since it is very A:T base pair-rich.

**Table 4:** G:C bonds/bp

Sample	Number of G:C bonds	G:C bonds/bp
Breakpoint		
Type C	64	0.26
Human Var.#3	6	0.05
Region		
Chimp Var.#3		
Region	3	0.028
^a^Random	1	0.014

^a^From Additional file 6: Figure S5

Although approaching a breakpoint hot spot stem loop (Type C) structure, the human Variable Region #3 secondary structure lacks important signatures of Type C and other PATRR stem loops. Thus, a further maturation is needed to form a structure that is more analogous to a PATRR.

Random sequences of the same length and A + T content as the human Variable Region #3 stem loop 2 fold into imperfect stem loops with significant numbers of “mini-stem loops” protruding from the main stem (e.g., see Fig. 3b). A total of forty random sequences were generated and analyzed for secondary structure. They display widely different types of structures, but importantly, seven out of the forty random sequences display very long stem loops (Additional file 5: Table S1). On the other hand, these structures show many imperfections in stems with bulged and looped out bases and mini-stem loops. One stem loop from a random sequence that best simulates a PATRR-type structure is 123 bp in length (Additional file 6: Figure S5, stem loop 1)**.** Thus, there is a probability that random mutations may play a role in building the secondary structures seen in the Variable Regions.

### 10,000 bp unit, translocation breakpoint inserts and RNA motifs

**Fig. 6:**
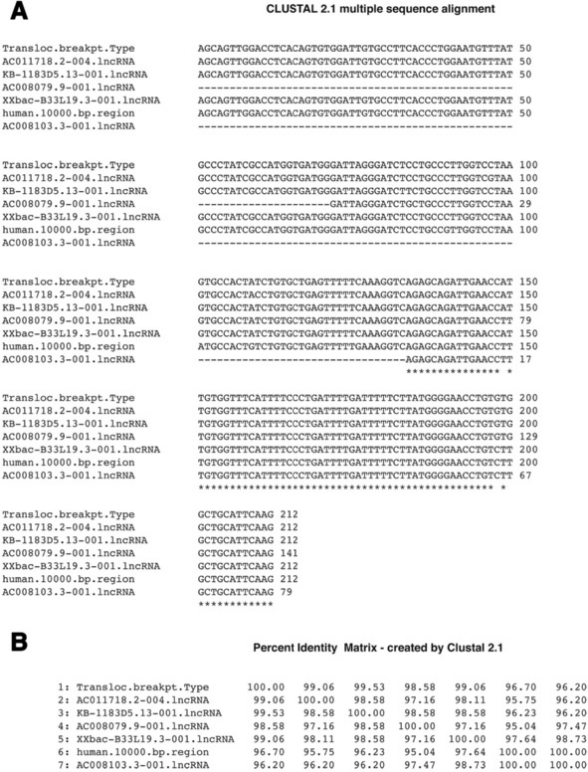
**a** Alignment of exon 1 sequences from lncRNAs, translocation breakpoint flanking sequence and the 10,000 bp unit. Nucleotide sequences of RNA exon 1 samples taken from transcript numbers shown on the Vega/Sanger website: http://vega.sanger.ac.uk/ or the Ensembl site: http://useast.ensembl.org/. The segment of the 10,000 bp unit chosen for the exon sequence alignment (positions 3993–4204) is from a previous breakpoint insert in this region. This exon sequence aligns in the reverse complement in the 10,000 bp unit. **b** Percent identities. Sequence alignment and percent identities is by Clustal W2 (www.ebi.ac.uk/Tools/msa/clustalw2/)

**Fig. 7:**
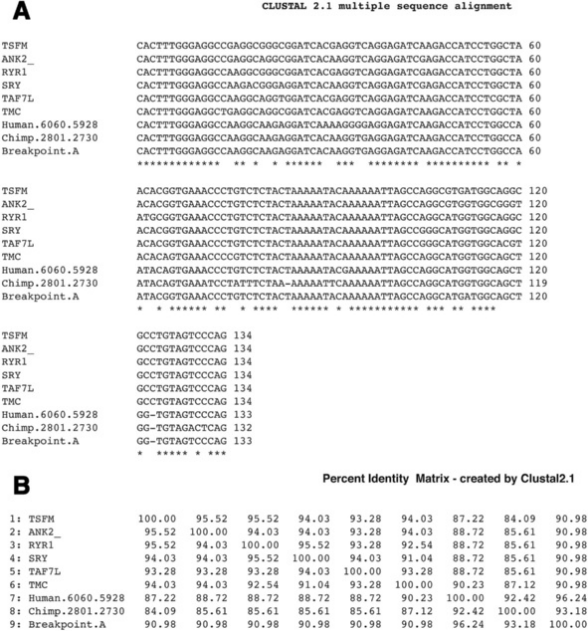
**a** Intron sequence alignment of protein genes, translocation breakpoint flanking sequence, the 10,000 bp unit and chimpanzee homologous region. Genomic positions of human and chimpanzee sequences are shown and are the reverse complement. Protein genes are shown in NCBI Gene Bank website: (http://www.ncbi.nlm.nih.gov/). TMC, transmembrane channel-like 1 , RefSeqGene on chromosome 9; RYR1, ryanodine receptor 1 (skeletal) (RYR1), RefSeqGene on chromosome 19; TAF7L, TAF7-like RNA polymerase II, TATA box binding protein (TBP)-associated factor, 50 kDa, RefSeqGene on chromosome X; TSFM, Ts.translation elongation factor, mitochondrial (TSFM), RefSeqGene on chromosome 12; SRY, (sex determining region Y)-box 5 (SOX5), RefSeqGene on chromosome 12; ANK2, ankyrin 2, neuronal (ANK2), RefSeqGene (LRG_327) on chromosome 4. Sequence alignment is by Clustal W2 (www.ebi.ac.uk/Tools/msa/clustalw2/). **b** Percent identities

RNA motifs that reside on the 5′ flanking region of the breakpoint Type A sequence (Additional file 4: Figure S4) are found in multiple locations in the 10,000 bp chromosomal segment as a result of breakpoint insertions in the segment (Fig. 1b). These motifs consist of an exon sequence, exon 1 that is found in multiple lncRNAs transcripts (Fig. 6) and a partial sequence of an intron found in several different mRNAs primary transcripts (Fig. 7). The translocation breakpoint sequence appears to be a conduit for spreading these RNA motifs.

An analysis of base pair changes in the exon sequence alignment from lncRNAs (Fig. 6) shows that out of 212 bp, one bp change (position 101) occurs specifically in the human 10,000 bp region but none occur in the translocation breakpoint sequence, whereas other changes occur amongst the lncRNAs themselves. To a first approximation, this may indicate that the translocation breakpoint exon sequence is the original source of exon sequences found in the lncRNAs. The exon sequence found in the human 10,000 bp region shown in Fig. 6 is present in the reverse complement and is at positions 3993–4204, which is in a breakpoint sequence insert region that is not present in the comparable chimpanzee region.

The mRNA intron sequence fragment (133 bp), present in the breakpoint flanking sequence is in both the human (positions 5928–6060) and chimpanzee (2801–2730) genomic regions. This sequence is found repeated in six different protein genes at ~91 % identity compared to the breakpoint sequence (Fig. 7a, b). The intron motif (here termed intron #1) is also found repeated many times in different introns of individual mRNAs, e.g., intron #1 motif is repeated 41 times in the ankyrin 2, neuronal (ANK2) gene (NCBI accession NG_009006). What stands out in the intron sequence alignment is the deletion at position 123 found only in the translocation breakpoint sequence, the human 10,000 bp sequence and the homologous chimpanzee sequence (Fig. 7a). A deletion may have occurred in the breakpoint Type A sequence before incorporation into the chimpanzee genomic region and before branching of the human species. The intron #1 sequence may have a common molecular function as it is abundantly found in several mRNAs and in multiple introns within an mRNA, however, it does not exhibit a special secondary structure or display a significant open reading frame.

### Phylogenetically conserved region

Sequences of positions 5757–9694 of the human 10,000 bp segment are highly conserved between human and chimpanzee with 97 % identity over 3937 bp of the human sequence. This raises the question as to why this non-protein coding region is so well conserved. However, there are two mRNA intron sequences in this region, one of which originates from a breakpoint flanking sequence insertion. The region also displays a high nt sequence identity to an lncRNA exon (exon 4) (Fig. 1b).

One intron (intron #1), is a fragment of an mRNA intron and is at positions 5928–6060 of the 10,000 bp unit; this has been discussed above. The sequence is conserved between human and chimpanzee to ~92 % and slightly less between human and the six mRNA introns (~87 %-90 %) (Fig. 7b).

**Fig. 8:**
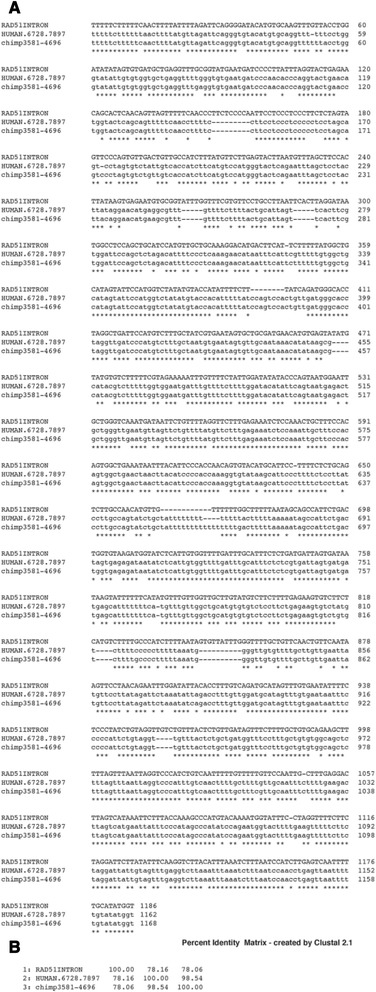
**a** Alignment via Clustal Omega of nt sequences from RAD51 intron [19], human 10,000 bp unit (positions 6728–7897) and the homologous chimpanzee region (positions 3581–4696). **b** Percent identity between the three sequences

A second intron sequence (intron #2) is at positions 6670–7897 of the human 10,000 bp segment and is highly conserved between human and chimpanzee sequences (99 %) (Fig. 8). This intron is found in the RAD51 paralog B (RAD51B) mRNA. The protein is a member of the RAD51 family proteins involved in DNA repair [23]. There is an identity of 78 % between RAD51B mRNA intron and both the human and chimpanzee sequences, but the intron sequence is not found in the translocation breakpoint flanking sequence. There are multiple copies of this intron within the RAD51B mRNA itself, but the sequence is only moderately conserved within the RAD51B mRNA (75 %-80 %). The sequence is found in other mRNAs as well such as that of uracil phosphoribosyltransferase (FUR1) homolog (S. cerevisiae) (UPRT) with an identity of 83 %. Thus this intron, found in several mRNAs and in the 10,000 bp region, is well conserved between humans and chimpanzee; the sequence seems to have been frozen with time between primates and humans (Fig. 8a, b).

Within the phylogenetically conserved region of the 10,000 bp unit, at human positions 8161–9421 there is a 1260 bp sequence that has an identity of 97 % with a segment of a Vega annotated lncRNA gene, AP003900.6 ENSG00000271308 (Chr. 21: 11,169,720-11,184,046). This 1260 bp sequence, found in the 10,000 bp unit includes the last exon sequence (exon 4) and short segments of flanking intron sequences of the AP003900.6 lncRNA gene. Exon 4 is also well conserved in the homologous chimpanzee region. This exon sequence adds to the variety of RNA motifs found in the 10,000 bp unit.

Positions 9423–9694 (272 bp) are well conserved between human, chimpanzee and other primates such as Rhesus Macaque but this sequence has similarities to a LINE1 element as determined by RepeatMasker [http://www.repeatmasker.org/cgi-bin/WEBRepeatMasker].

It should be noted that Alu SINE elements have been previously found at breakpoint regions and these are associated with gene repeats within LCRs on 22q11.2 [24, 25]. No duplication of the highly conserved sequences (positions 5757–9694) of the human 3′ region of the 10,000 bp unit has been observed in human chromosome 22, but one copy of the 10,000 bp sequence containing the entire conserved sequence is present in each of chromosomes 13 and 21 (ND, unpublished data).

### 3′ end of 10,000 bp segment

**Fig. 9:**
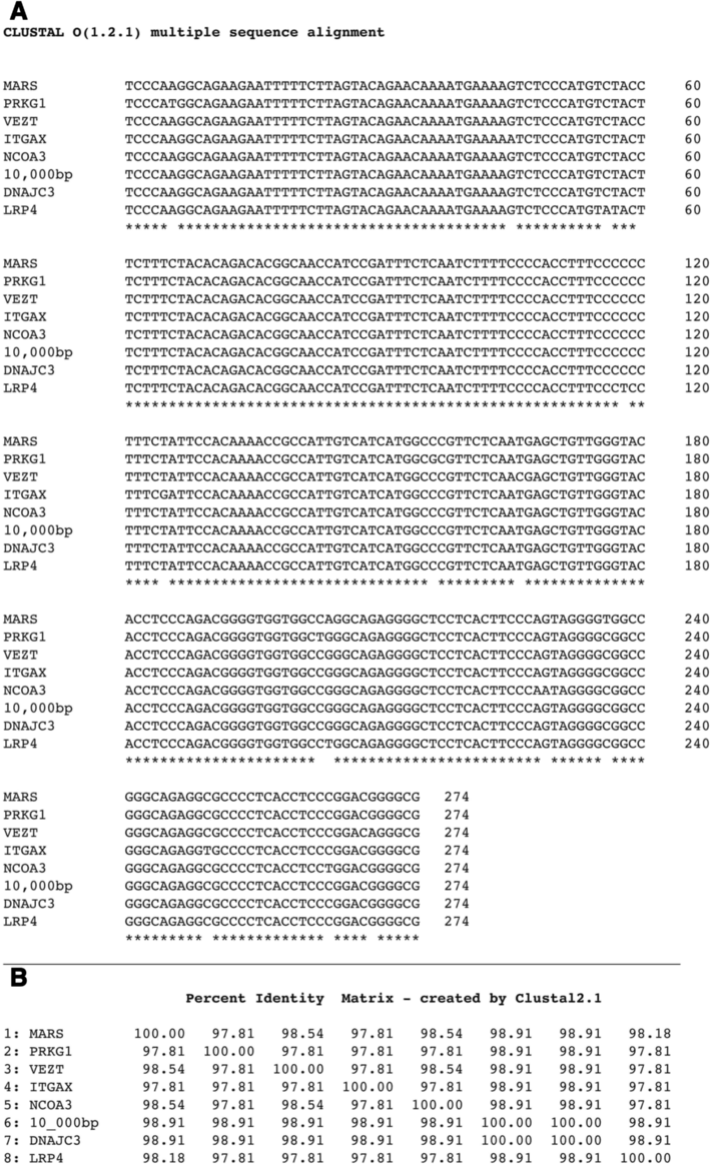
**a** Alignment using Clustal Omega of nt sequences from seven protein gene introns and the 10,000 bp unit (positions 9700–9974) (only seven of the the protein gene introns that have been detected are shown). Protein genes: PRKG1 protein kinase, cGMP-dependent, type I (PRKG1), RefSeqGene on chromosome 10; NCOA3, nuclear receptor coactivator 3 (NCOA3), RefSeqGene on chromosome 20; ITGAX, integrin, alpha X (complement component 3 receptor 4 subunit) (ITGAX), RefSeqGene on chromosome 16; MARS, methionyl-tRNA synthetase (MARS), RefSeqGene on chromosome 12; LRP4, low density lipoprotein receptor-related protein 4 (LRP4), RefSeqGene on chromosome 11; DNAJC3, DnaJ (Hsp40) homolog, subfamily C, member 3 (DNAJC3) gene; VEZT, vezatin, adherens junctions transmembrane protein (VEZT), RefSeqGene on chromosome 12;. **b** Percent identity between 10,000 bp unit and seven intron segments

A fragment of a third mRNA intron (intron #3) is present at the 3′ end of the human 10,000 bp region, positions 9700–9974 bp (274 bps), but this sequence is not present in the translocation breakpoint sequence and does not show significant identity with the chimpanzee sequence. The sequence is present in many different protein genes that includes low-density lipoprotein receptor-related protein 4 (LRP4) (Fig. 9a). There is a very high identity between the 10,000 bp unit and the seven-intron sequences shown (99-100 %) (Fig. 9b). There appears to be no obvious pattern in substitutions between the mRNA intron sequences themselves or with the 10,000 bp unit.

### Translocation breakpoint sequences and linked A + T-rich regions are present in different locations of 22q11.2

**Fig. 10:**
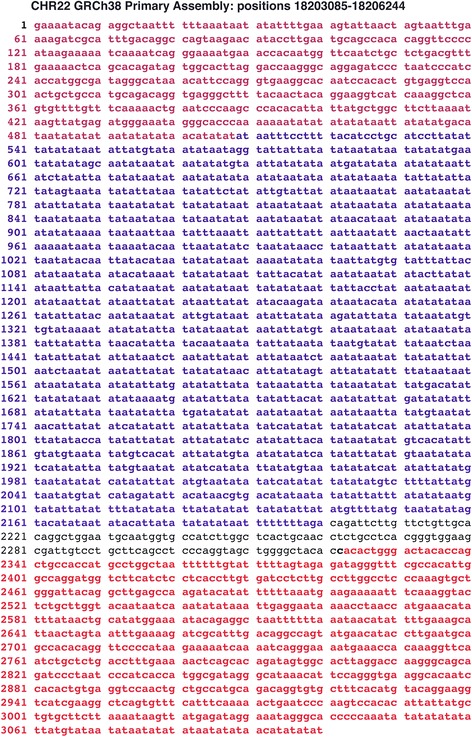
Nucleotide sequence from Homo sapiens chromosome 22 (GRCh38 Primary Assembly, coordinates 18203085–18206244. Shown in red highlighted lettering are sequences that represent segments of the translocation breakpoint Type A sequence. The top 1–507 positions in the figure have an identity of 97 % with positions 310–814 on translocation breakpoint Type A but is present in the reverse complement. The bottom highlighted region, positions 2323–3100 in the figure have an identity of 96 % with position 310–1088 of translocation breakpoint Type A sequence, also in the reverse complement. The sequence highlighted in blue (positions 509–2200) represents the high A + T (93 %) region. The short unhighlighted sequence in black letters has not been characterized

Besides its presence in the 10,000 bp sequence, the unusual pattern of translocation breakpoint sequences linked to high A + T regions is also found in other regions of 22q11.2. In one example, a region was found in Chr22 (positions 18203085–18206244) that has sections of very high identity to the breakpoint type A sequence and the breakpoint sequences are linked to variable A + T-rich sequences (93 % A + T), a pattern similar to that of the 10,000 bp variable region. Figure 10 shows two breakpoint sequences (highlighted in red) that straddle a high A + T sequence (highlighted in blue).

In a similar vein to the 10,000 bp region, the high A + T-containing region has 58 copies of the sequence TATAATATA, whereas twenty-five random sample sequences of the same length and A + T content average 1.56 copies. The p-value in this case is also <0.0001.

Breakpoint sequences have been extensively duplicated in 22q11.2, and we hypothesize that they may carry information to form adjacent highly biased A + T regions that mutate extensively and are found in several regions of 22q11.2.

## Discussion

Aside from protein coding regions, most of the human genome has not been defined in terms of function, although it is fascinating that much of the genome is transcribed into RNA and a large number of non-coding RNA genes have been characterized [26, 27]. In the analysis of a minute part of chromosome 22, the 10,000 bp region, there may be functions independent of regulation, expression of protein products, or RNA transcripts. With respect to RNA transcripts, no transcript with 100 % identity to annotated lncRNAs has yet been shown to originate from the 10,000 bp region (L. Wilming, Welcome Trust Sanger Institute, personal communication). However, we show here that the 10,000 bp unit has segments with highly variable sequences that appear to be reserved for biased genetic mutational processes. We hypothesize that these may lead to formation of molecular functions, e.g., PATRR formation. On the other hand, the highly conserved sequence at the 3′ half of the 10,000 bp region may be reserved for the storage of RNA motifs. The pattern, i.e., 5′ end segment reserved for mutations and 3′ end of 10,000 bp containing phylogenetically conserved sequences, is conserved between chimpanzee and human genomes, which supports the idea of functional roles.

The three sequence Variable Regions of the 10,000 bp unit, consisting of approximately 1000 bp each have mutations that may be viewed as a trial and error processes with a strong partiality to maintaining a very high A + T content and formation and maintenance of many copies of the TATAATATA motif. Conceptually, this can be viewed as a process analogous to a “biased random walk” as in chemotaxis [28–30]. The analogy is that some mutations eliminate a TATAATATA unit in the human segment (e.g., see Fig. 2) but a larger number of mutations are involved in forming the unit (Table 1). As an accurate alignment of sequences within most of the Variable Regions is not possible due to the presence of low complexity sequences, we can not determine the specific number of TATAATATA units added by base pair mutations or by sequence addition, and the number lost relative to the chimpanzee sequence. Although a function for the conserved TATAATATA motif in translocation has not been determined, what is shown here is a strong bias towards forming and/or maintaining the motif with significantly more copies created in the human genome relative to that in the chimpanzee. Cairns and co-workers [31] originally proposed the concept of non-random or directed mutations. Recently, Martincorena, Luscombe and coworkers [32, 33], by using phylogenetic and population genetic methods provide evidence for non-random mutation rates. The proposal here on Variable Region mutations is consistent with these previously formed concepts, but as a biased random process. A major question remains, and that is what is the molecular mechanism that drives the mutational process towards this putative biased random mutational process? The variable regions are all linked to translocation breakpoint flanking sequences, thus, it is possible the information lies in these flanking sequences, or in the variable sequences themselves with perhaps a mechanism analogous to microsatellite replication [34].

The translocation breakpoint 5′ end sequence flanking the stem loop hot spot seems to be a major player in carrying and spreading exon and intron motifs in the genome. Jacob intuitively suggested decades ago that building blocks are used over and over in biological processes to create new functions [35]. What we see here are exon and intron sequences that are used by many different genes and are found in different and multiple lncRNAs and mRNAs, respectively.

Some intron segments are found repeated in different introns of the same mRNA, such as in ryanodine receptor 1 (skeletal) (RYR1) mRNA that has 36 repeats of the same sequence. This intron sequence is also is carried by the breakpoint sequence. A question is why do many different genes carry the same sequence? What may be special about these intron and exon sequences is that they perform a common function in different RNAs, or within the same mRNA.

## Conclusions

Sequence Variable Regions at the 5′ end of the 10,000 bp non-coding segment of 22q11.2 show a biased random pattern of mutations that produces a very high A + T content, sequences that fold into long stem loops, and a high abundance of the nine nucleotide sequence TATAATATA; these Variable Sequence Regions are invariably linked to translocation breakpoint Type A 5′ flanking segments. We hypothesize that with further stem loop development these may function in DNA translocation. The 3′ half of the 10,000 bp region consists of sequences that are highly conserved between human and chimpanzee genomes, and this region contains various RNA motifs: both protein gene intron fragments and lncRNA gene exons, and some are carried by translocation breakpoint flanking sequences. As these motifs are well conserved between the two primate species and are found in multiple lncRNA or protein genes, they may be stored for future use in synthesis of new molecular functions. To our knowledge this is the first observation of translocation breakpoint sequences carrying and spreading RNA motifs and we suggest they may be reservoirs for use in formation of new lncRNA and protein genes.

In conclusion, this study defines a genomic region that is proposed to function independently of encoding protein or RNA genes, i.e., sections reserved for biased mutations and the storage of RNA motifs.

## Methods

### Genomic sequence searches

To find DNA nt sequences corresponding to the 10,000 bp human sequence (Homo sapiens chromosome 22, GRCh38 Primary Assembly) and chimpanzee homologous sequence (Pan troglodytes chromosome 22, Pan_troglodytes-2.1.4), the NCBI-Blast program was used (http://blast.ncbi.nlm.nih.gov/Blast.cgi?CMD=Web&PAGE_TYPE=BlastHome).

The human and chimp blast pages were employed.

### Alignment methods

Alignment of global DNA sequences was by one of three methods: 1. EMBL Clustal W2 (http://www.ebi.ac.uk/Tools/msa/clustalw2/) [14]; 2. ExPaSy LALIGN (http://embnet.vital-it.ch/software/LALIGN_form.html) [15]; and 3. Emboss Needle: (http://www.ebi.ac.uk/Tools/psa/emboss_needle/nucleotide.htm)

EMBOSS: the European Molecular Biology Open Software Suite [16]. The default settings were used in each case. All three programs gave a similar overall alignment between human and chimpanzee 10,000 bp sequences, however, it was not possible to obtain a consistent alignment within Variable Regions with the exception of some small segments.

Alignment of two or more sequences with reverse complement identities was by NCBI-Blast Align Sequences Nucleotide BLAST (http://blast.ncbi.nlm.nih.gov/Blast.cgi?AGE_TYPE=BlastSearch&BLAST_SPEC=blast2seq&LINK_LOC=align2seq ). The NCBI Blast program was used to find exon and intron homologs to those found in the translocation breakpoint flanking sequence and 10,000 bp unit.

### LncRNA searches

Search for lncRNAs in chromosome 22 22q11.2 region was with three web servers: 1. Vega/Sanger website (http://vega.sanger.ac.uk/Homo_sapiens/Location/Chromosome?r=22), 2. Ensembl/Wellcome Trust/Sanger Institute/European Bioinformatics Institute: (http://useast.ensembl.org/Homo_sapiens/Location/View?db=core;g=ENSG00000197421;r=22:18782111–18812514;t=ENST00000430306), and 3. UCSC Genome Broswer: (http://genome-euro.ucsc.edu/cgi-bin/hgTracks?db=hg19&position=chr22%3A18890337-18900336&hgsid=198277878_3neHZpMdbjv5a37IhFXAN7DDTgGb).

### Generated random sequences

Random sequences were generated by Molbiol.ru and adding the nucleotide length and % GC-content: (http://molbiol.ru/eng/scripts/01_16.html) Reverse complement sequences were by using the Sequence Manipulation Suite (http://www.bioinformatics.org/sms2/rev_comp.html).

### DNA secondary structure modeling

Folding of DNA sequences for secondary structure features was with the mFold Web Server: (http://www.bioinformatics.org/sms2/rev_comp.html). Standard conditions (default setting) of folding temperature, ionic conditions and constraint values as were employed [36, 37]. Structures 1 which display the lowest delta G values were chosen.

### Alu SINE and LINE-1 searches

RepeatMasker [http://www.repeatmasker.org/cgi-bin/WEBRepeatMasker] was used to determine presence of Non-LTR retrotransposable elements Alu and LINE-1 in the 10,000 bp region of 22q.11.2.

### *p*-value determinations

*p*-values for the determined copy number of TATAATATA units in a chromosomal segment vs. the copy numbers of random generated sequences was determined by a conservative nonparametric test, Wilcoxon’s signed rank test [22].

## Additional files

Additional file 1: Figure S1. Alignment of sequences from Homo sapiens chromosome 22 (GRCh38 Primary Assembly, coordinates:18890337–18900336) and Pan troglodytes chromosome 22, Pan_troglodytes-2.1.4, (coordinates: 17300774 to 17307562). Alignment was by Clustal W2. Breakpoint sequences are missing in the chimpanzee, although some small segments are present, e.g., the eighteen nucleotide sequence in the chimpanzee (positions 1367–1384) that are identical to the human breakpoint sequence. (PDF 30 kb) Additional file 2: Figure S2. Secondary structure that yields high frequency of translocation. Nucleotide sequence is from PATRR Type C, accession AB 538237. Secondary structure determined by DNA mfold program [36]. (JPEG 48 kb) Additional file 3: Figure S3. Example of a secondary structure that yields very low frequency of translocation. Sequence is from accession AF 391128 on chromosome 11. Secondary structure determined by DNA mfold program [36]. (JPEG 164 kb) Additional file 4: Figure S4. Translocation breakpoint and flanking sequences, Type A from Accession GenBank: AB261997.1. Color code: red, intron sequence; blue, exon 1 sequence; yellow, translocation breakpoint hot spot stem loop sequence (PATRR). (JPEG 502 kb) Additional file 5: Table S1. Folded random sequences- stem bp, lengths. (TIFF 303 kb) Additional file 6: Figure S5. Longest and best formed stem loop (stem loop 1) from a sample of 40 random sequences. Random sequence of 1393 nt, 8.3 % G + C. Sequence folded by mfold [36]. (DOCX 53 kb)
