# Supplementary material for: Complexity of a small non-protein coding sequence in chromosomal region 22q11.2: presence of specialized DNA secondary structures and RNA exon/intron motifs
Source: BMC Genomics. 2015 Oct 14;16:785. doi: 10.1186/s12864-015-1958-6 (PMC4607176; doi:10.1186/s12864-015-1958-6)
Supplement: Additional file 1: Figure S1. — Alignment of sequences from Homo sapiens chromosome 22 (GRCh38 Primary Assembly, coordinates:18890337–18900336) and Pan troglodytes chromosome 22, Pan_troglodytes-2.1.4, (coordinates: 17300774 to 17307562). Alignment was by Clustal W2. Breakpoint sequences are missing in the chimpanzee, although some small segments are present, e.g., the eighteen nucleotide sequence in the chimpanzee (positions 1367–1384) that are identical to the human breakpoint sequence. (PDF 30 kb) [file 12864_2015_1958_MOESM1_ESM.pdf]

```
CLUSTAL 2.1 multiple sequence alignment


human.10_000bp      TGCAGATGTAAAAGGAAATTATATATATATATATATATATATAATATATATACGTATATA 60
chimpanzee          ------------------------------------------------------------


human.10_000bp      TTATGAAGATATATGTATATATATTATATATAATTATATATATAATATATATATATAATTTG 120
chimpanzee          ------------------------------------------------------------


human.10_000bp      GGAATTTGGGAATAAATTGTATCCCAACTCACACTGGGACTACACCAGCTGCCACCATGC 180
chimpanzee          ------------------------------------------------------------


human.10_000bp      CTGGCTAATTTTTCGTATTTTTAGTAGAGACAGGGTTTCACTGAATTGGCCAGGATGGTC 240
chimpanzee          ------------------------------------------------------------


human.10_000bp      TTGATCTCCTCACCTTGTGATCCTCTTGCCTTGGCCTCCCAAAGTGCTGGGATTACAGGC 300
chimpanzee          ------------------------------------------------------------


human.10_000bp      CTGAGCCAAGATACATATTTTTTAAAGGAAGAAAAATTTCAAAGGTACTCTGTTTGGTAC 360
chimpanzee          ------------------------------------------------------------


human.10_000bp      AATAATCAAATATATAAATTGAGGAATAAAACATAACCACGAAACATATTTATAACTGCA 420
chimpanzee          ------------------------------------------------------------


human.10_000bp      TATGGAAAATACAGAGGATAATTTTTTAAATAACATATTTTGAAAACCTTAACTAGGAAT 480
chimpanzee          ------------------------------------------------------------


human.10_000bp      TTGAAAAGATCGCATTTGACAGGCCAGTATGAACACAACTTGAACGCAGCAAGACAGGTT 540
chimpanzee          ------------------------------------------------------------


human.10_000bp      CCCCATAAAAAAATCAAACACAGGGAAAATGAAACCACAAAGTTTCAATCTGCTCTGACC 600
chimpanzee          ------------------------------------------------------------


human.10_000bp      TTTGAAAAACTCAGCACAGACAGTGGCACTTAGGACCAACAGCAGGAGATCCCTAATCCC 660
chimpanzee          ------------------------------------------------------------


human.10_000bp      ATCACCATGGCGATAGGGCATAAACATTCCAGGGTGAAGTCACAATCCACATTGGGAGGT 720
chimpanzee          ------------------------------------------------------------


human.10_000bp      CCAACTGCTGCCAGGCAGACAGGTGTGCCTTTACATGTACAGGAAGGTCATTGAAGGCTC 780
chimpanzee          ------------------------------------------------------------


human.10_000bp      AGTGTTTTGTTTCAAAAACTGAATCCCAAGACCAAACATTGTTATGCTGTGCTTCTTAAA 840
chimpanzee          ------------------------------------------------------------


human.10_000bp      ATAAGTTATGAGATGGGAAAGAGGGCACCCCAAATATATATATATAATTATATATAATAT 900
chimpanzee          ------------------------------------------------------------


human.10_000bp      AATATATATAATATACATAATTATGTATAATATAATATATATAATATACATAATCTCCTT 960
chimpanzee          ----------ATATACATAATTATATATAATTTAATATATATAACAGATACAATTTCCTT 50
                              **************.******:************ * * * *** *****

human.10_000bp      TTACATCCTGCATCCTTATATTATATATAATATATTATATATAATAATATATAATAATAT 1020
chimpanzee          TTACATCCTGCATCATTATAATCTATATATTATCTGCAATATAATAGTAAATAAT----- 105
                    **************.*****:*.******:***.* .:********.**:*****     

human.10_000bp      ATAATTATTATATATATAATATATACAATTATATATGATTATATAATTGTATATAATTAT 1080
chimpanzee          --------------TTTAATATATAATATAATATATAAT--------------------T 131
                                  *:*********.:**:******.**                    *

human.10_000bp      ATATATAATATAATAATATATAATATCTATTATTATATTATATATAATATAATATTATAA 1140
chimpanzee          ATATATACTATAACAATATATTCTATAGAATAT-----------------------ATAT 168
                    *******.***** *******:.***. *:***                       ***:

human.10_000bp      TATATATCATATATTATAATATTATATGTAATATAATATAATATCATAATATATAATATA 1200
```

```
chimpanzee          TATATATCATATAT--------TATATGATATATAATATATTA---------AATATATA 211
                    **************        ******::**********:**         *::*****

human.10_000bp      TACTATAATATTATATATAATATAATATATTGCATAATATATAATATATACAATATATTA 1260
chimpanzee          TTATATAATATTACATATGATAT----------ATAATATATATTCTATATAATATATCA 261
                    *:.********** ****.****          **********:*.**** ******* *

human.10_000bp      TGTATAATATATAATATATAATATTATATATAATATGTCATACTATAATATATATTACAA 1320
chimpanzee          G----AATATAATATATATATAATAATATATGTAATAT----------------------A 294
                         ******::**********:*****.******                      *

human.10_000bp      TATAATATATATAATATTACATATATAATATAAAATATTACATAATATATAATAATAATA 1380
chimpanzee          TATAATATATATTATATTTTATATAGAATATGCAATATTATATAATATATTTAATATATA 354
                    ************:*****: ***** *****..******* *********:::*:::***

human.10_000bp      TAATATAATATGTAATATTATATAATATGTAATAATATAATATATAATATATTATGATAT 1440
chimpanzee          TTATATAATAT----------------------ATTTAATATATATTATAT-------- 383
                    *:*********                      **:*********:*****

human.10_000bp      ATATAATATAATATATTATATCTTATGATATATTATATATAATATAATATATGATAATAT 1500
chimpanzee          ------------------------ATAATATAATACATATAATAAAATATATAACATTAT 419
                                            **.*****:** ********:*******.* *:***

human.10_000bp      ATAATATATTATGATACATTATATATAATATAATATATGATATAAGATATTATATATGAT 1560
chimpanzee          ATAATATAATATATAACAATATATAATACAAAATA-ATAATGTATGATATATTAAAT--A 476
                    ********:***.::***:******::* *:**** **.**.**:*****::**:**  :

human.10_000bp      ATATGATGTAATATATATAATGTATAATATATAATATATATAATATGTAATATATATTAT 1620
chimpanzee          TTATGATATATTACATATAATGTAATATAATATAATTATGTGTAATATAATATATAATAC 536
                    :******.**:** **********::***:::*::***.*.::**.*********:**

human.10_000bp      ATATTATATATTATATGTAATATATATTATATATTATATATTATATGTAATATGTATTAT 1680
chimpanzee          AT-------------TAAAATATATATCTTATATTTTATAAAAGATGTCATAT--ATGAT 581
                    **             *.:********* :******:****::* ****.****  ** **

human.10_000bp      ATATTATATGTAATATATATTATATATTATATGTAATATATATTATATATTATATGTAAT 1740
chimpanzee          ATATAAGATATCATAAATATTATATATAATGTATCATATATTATATATAATATATG---- 637
                    ****:* **.*.***:***********:**.*.*.******::******:******

human.10_000bp      ATATATTATATATTATATGTAATATATATTATATATGATATATAATATGACATTATATAT 1800
chimpanzee          ATATATTATATGTTATATATAATACATTATATATAATATATATCTTATAT------------ 685
                    ***********.******.***** **::******: ****.*:****

human.10_000bp      GATATGACATATAATATATATTATATATTACATGTATACTACATATATTATATATGGTAT 1860
chimpanzee          ------------------------------------------------------------

human.10_000bp      ATAATATATACTATATATAATGTATATAATAGATATAATATATAATATATAATACATTTT 1920
chimpanzee          ------------------------------------TATATATCATATATCATACATTAT 709
                                                        :******.******.*******:*

human.10_000bp      ATATAATATATAATATATAATATATATAATATATAATATATATTATATATAATATAAAAA 1980
chimpanzee          ATATAATATATCATATATCATATATTATATAT---------------------------- 741
                    ***********.******.******:::****

human.10_000bp      ATATATGTTATATAAAATATATATAATATATATTATATATATATAATTTTTTTGAGACAG 2040
chimpanzee          ------------------------------------------------------------

human.10_000bp      AGACTTTTTCTGTTGCACAGGCTGGAATGCAATGGCGCCATCTTGGTTCACTGCAACCTC 2100
chimpanzee          ------------------------------------------------------------

human.10_000bp      TGCCTCACGGGTTCAACTGATTGTCCTGCCTCAGCCTCCCAGGTAGCTGGGACTACACCA 2160
chimpanzee          ------------------------------------------------------------

human.10_000bp      CACTGGGACTACACCAGCTGCCACCATGCCTGGCTAATTTTTTGTATTTTTAGTAGAGAC 2220
chimpanzee          ------------------------------------------------------------

human.10_000bp      GGGGTTTCAGTATATTGGCCAGGATGGTCTTCATCTCCTCCCCTTGTGATCCTCTTGCCT 2280
chimpanzee          ------------------------------------------------------------

human.10_000bp      TGGCCTCCCATAGTGCTGGGATTACAGGCCTGAGCCAAGATACATATTTGTTAAAGGAAG 2340
chimpanzee          ------------------------------------------------------------

human.10_000bp      AAAAATTTCAAAGTTACTCTGCTTGTTACAATAATCAAATCTGTAAATTGAGGAATAAAA 2400
chimpanzee          ------------------------------------------------------------
```

```
human.10_000bp    CATAACCATGAAACATATTTATAACTGCATATGGAAAATACAGAGAATAATTTTTTAATA 2460
chimpanzee        ------------------------------------------------------------


human.10_000bp    ACATATTTTGAAAACATTAACTAGGAATTTGAAAAGATCGCATTTGACAGGCCAGTATGA 2520
chimpanzee        ------------------------------------------------------------


human.10_000bp    ACATACCTTGAATGCAGCAAGACAGGTTCCCCATAAGAAAAATCAAAATCAGGGAAAATG 2580
chimpanzee        -CATACATTATAT----------------------------------------------- 753
                   *****.**.:**

human.10_000bp    AAACCACAAAGGTTCAATCTGCTCTGACCTTTGAAAAACTCAGCACACACAGTGGCACTT 2640
chimpanzee        ------------------------------------------------------------


human.10_000bp    AGGACCAACGGCAGGAGATCCCTAATCCCATCACCATGGCGATAGGGCATAAACATTCCA 2700
chimpanzee        ------------------------------------------------------------


human.10_000bp    GGGTGAAGGCACAATGCACATTGGGAGGTCCAACTGCTGCCATGCAGACAGGTGTGCTTT 2760
chimpanzee        -------------------------------------------GCAGACAGGTGTGCTTT 770
                                                             *****************

human.10_000bp    TACATGTGCAGGAGGATCATTGAAGTCACAGTGTTTTGTTTCAAAAACTGAATCCCAAGC 2820
chimpanzee        TACATGTACAGGACAGTCATTGAAGGCTCAGTGTTTTGTTTCAAAAACTGAATCCCAAGC 830
                  *******.***** ..********* *:********************************

human.10_000bp    CCACACATTATTATGCTGTGCTTCTTAAAATAATTTATGAGATGGGAAATATGGCACCCC 2880
chimpanzee        CCACACATTATTATGGTGTCCTTCTTAAAATAAGTTATGAGATGGGAAATAGGGCACCCC 890
                  *************** *** ************* ***************** ********

human.10_000bp    CAAATATATATATATATAATTATATATAATATAATATATATAATAGATATAATTTCCTTT 2940
chimpanzee        CAAATATATATATACATAATTATATGTAATACAATATATAATACATATG----------- 939
                  ************** **********.***** ********::* * **.

human.10_000bp    TACATCCTGCATCCTTATATATATTACATTATATAAAATATAATAATATATAATTATATA 3000
chimpanzee        --------------------ATTATACATAATATAATATATATAATACATGTAATATATA 979
                                      **::*****:******:*****::*::.:: :*:******

human.10_000bp    TAATATAATATAATAATATAAAATATATATTACTATATTATATATAATATATAATATATA 3060
chimpanzee        TAACTTCCT-------TTTTCATCCTGCATCCTTCCATTATATATAATATATTATATATA 1032
                  *** :*..*       *:*:.*: .*. ** . *. ****************:*******

human.10_000bp    TTATATATTAAACAATACAATAACATATAATATATGTTATATATAATACTATATATAATG 3120
chimpanzee        TTATAAT--------------------------------AATATATAATAATATATAAT- 1059
                  *****::                               :*****::* :*********

human.10_000bp    TATAACATATATAATATTACCTATGATATATAATAATATATATTATGTATAATATATCAT 3180
chimpanzee        --TATCATATATAATATAACATAT----------------AATTATATATAATT----AT 1097
                    **:************:**.***                :*****.******:    **

human.10_000bp    ATTATAATATATATAATATAGTATATATAATATGTATTATATAATATCTAATATTATATA 3240
chimpanzee        ATATGATCATATATAATATAATATGTATAAT--------------------TATATAATG 1137
                  **:: *: ************.***.******                    ***:::**.

human.10_000bp    ATATATAATATATCATATATAATATATTATGATATATTATATATAATATAATACAATATA 3300
chimpanzee        ATATATATAATTACATCTATAATATAGTAAAATATATTGTATATTATAT-ATGATATATA 1196
                  *******::**::***.********* **:.*******.*****:**** **..:*****

human.10_000bp    ATATATTATATATTATGATACATTATATATAATATAATACAATAAAATATATTATATATT 3360
chimpanzee        ATATAGTAATATTTATTTTACATTATATATTATAT---ACAATACAATATATTACATATA 1253
                  ***** **::::**** :************:****   ******.********* ****:

human.10_000bp    ATGATACATTATATATAATATAATACAATAAAATATATTATATATTATGATATATAATAT 3420
chimpanzee        AT-------------------------ATATAATACATAATATATGATGTTCTATATTAT 1288
                  **                         ***:**** **:****** ***:*.****:***

human.10_000bp    GTAATAAAATATAATATAATTATATATAATATAATATATAACATATAATATTTATGATAT 3480
chimpanzee        G---TATAATATATTGAATATATAAAATATATATTAAATCATATATATTATATATAATAT 1345
                  *   **:******:*.:*::****:*::*****:**:**.* *****:***:***.****

human.10_000bp    TATATATATATAATTTTTTTGAGACAGAGACTTTCTGTTGCACAGGCCAGAGTGCAATTG 3540
chimpanzee        ------------------------------------------------------------


human.10_000bp    CGCCATCTTGGCTCACTGCAACCTCTGCCTCATTGGTTCAAGCGATTGTCCTGCCTCGGC 3600
chimpanzee        ------------------------------------------------------------
```

```
human.10_000bp          CTCCCAGGTAGCTGGGACTACACCACACGGGGACTACACCAGGTGCCACCATGCCTGGCT 3660
chimpanzee              ---------------------------------------------CCAT----------- 1349
                                                                     ***

human.10_000bp          AATTTTTGTATTTTTAGTAAAGACAGGGTTTCACTGTATTTGCCAGGATGGTCTTCATC 3720
chimpanzee              ------------------------------------------------------------

human.10_000bp          TCCTCACCTTGTGATCCTCTTGCCTTGGCCTCCCAAAGTGCTGGGATTACAGGCCTGAGC 3780
chimpanzee              ------------------------------------------------------------

human.10_000bp          CAAGATACATATTTGTTAAAGGTAGAAAAATTTCAAATGTACTCTGCTTGGTACAATAAT 3840
chimpanzee              ------------------------------------------------------------

human.10_000bp          CAAATATATAAATTGAGGAATAAAACATAACCATGAAACATATTTATAACTGCATATGGA 3900
chimpanzee              ------------------------------------------------------------

human.10_000bp          AAATATAGAGGATAATTTTTTAAATAACATATTTCAAAAGCATTAACTAGCAATTTGAAA 3960
chimpanzee              ------------------------------------------------------------

human.10_000bp          AGATCGCATTTGACAGGCCAGTATGAACATACCTTGAATGCAGCAAGACAGGTTCCCCAT 4020
chimpanzee              ------------------------------------------------------------

human.10_000bp          AAGAAAAATCAAAATCAGGGAAAATGAAACCACAAAGGTTCAATCTGCTCTGACCTTTCA 4080
chimpanzee              ------------------------------------------------------------

human.10_000bp          AAAACTCAGCACAGACAGTGGCATTTAGGACCAACGGCAGGAGATCCCTAATCCCATCAC 4140
chimpanzee              ------------------------------------------------------------

human.10_000bp          CATGGCGATAGGGCATAAACATTCCAGGGTGAAGGCACAATCCACACTGTGAGGTCCAAC 4200
chimpanzee              ------------------------------------------------------------

human.10_000bp          TGCTGCCATGAAGACAGGTGTGCTTTTACATGTACAGGAAGGTCATTGAAGGTTCAGGGT 4260
chimpanzee              ------------------------------------------------------------

human.10_000bp          TTTATTTCAAAAACTGGATCCCAAGCCCACACATTATTATGCTGTGCTTCTCAAAATAAG 4320
chimpanzee              ------------------------------------------------------------

human.10_000bp          TTATGAGATGGGAAATAGGGCACCCCCAAATATATATATATATATTTATAATTATATATA 4380
chimpanzee              --------------------------------------------TATATTATTACATTAA 1365
                                                                    *:***:**** **::*

human.10_000bp          ATATAATATATAATATATATAACACATATATAATTTCCTTTTACATCCTGAATCCTTATA 4440
chimpanzee              GTATAATATATAATATATA------ATATATGATT-----------------ACATTATA 1402
                        .******************      ******.***                 :*.*****

human.10_000bp          TTATATATAACATATTATATAGAAGATATATAATATATTATTTTGTACATAATATAATAT 4500
chimpanzee              TATTATATTATATATTATATTAGA---------------------TACATAATACAATAT 1441
                        *::*****:* *********:..*                     ********* *****

human.10_000bp          ATATAATTATATCTAAATATATAATTATATATAATTATATCTAAATATATAATTATATAT 4560
chimpanzee              ATAATATTATAT---AATATATAATAATAAATAT---------AATATATAAG------- 1482
                        ***::*******   **********:***:***:         *********

human.10_000bp          AATTATATCTAAATATATAATTATATATAATTATATCTAAATATATAATTATATATAATT 4620
chimpanzee              --------------ATATGATTATATATAAT---ATAATATAATATAATTATATATAAT- 1524
                                      ****.************   **.::*::*****************

human.10_000bp          ATATCTAAATATATAATTATATATAATTATATCTAAATATATAATTATATATAATTATAT 4680
chimpanzee              -------ATAATATAATCATTTGTAAT----------------ATAATATATATTATATT 1561
                               *::******* **:*.****                **:*******:*::::*

human.10_000bp          CTAAATATATAATTATATATAATTATATCTAATATATAATATATTATGCTATATTATACA 4740
chimpanzee              ATATATAATATATCATATATAATAATATTTCATATATTATATAATAG---ATATCATACA 1618
                        .**:***::::** *********:**** *.******:*****:**    **** *****

human.10_000bp          TAATATAATATAATGATATATAGTATAATATAATATATATCATATATATCATATATTATA 4800
chimpanzee              TTAGAGATTAT----------------ATATAATATATATCATATGTTATATATAATATT 1662
                        *:* * *:***                ******************.*:: *****:***:
```

```
human.10_000bp      TATCATTATATTATATTTAATAGATATCATATATTATATATAGTATATATCATATATTAT 4860
chimpanzee          TATCAT-CTATTATATATAATGTATATCATGTTTTATATATAACATATATCATGTATTAT 1721
                    ****** .********:****. *******.*:*********. *********.******

human.10_000bp      ATATAATATGTAACATATAATATATAATATATATCATTCATTATACTATATATTATATAT 4920
chimpanzee          ATATTATATATCATGTATTATATATAATATATATCAT-----ATATTATATATAATAGAG 1776
                    ****:****.*.* .***:******************     *** *******:*** *

human.10_000bp      AATATATATCATATATTATATACATAATATATGATATATATTATATATAATACATGGTAT 4980
chimpanzee          GAAATATATTATATATATTATATTTAATATATGATACATATTATATAATATACAT--AAT 1834
                    .*:****** ******::**** :************ *********::******  :**

human.10_000bp      ATATTATATATAATATATGGTATATATTATATATAATACATGGTATATATTATATGTAAT 5040
chimpanzee          ATATTATAAATAATGTAT---ACATTATATATTATATACGTGATATATTCAATAT---AT 1888
                    ********:*****.***   * **::*****::****.**.*****: :****   **

human.10_000bp      ACATGGTATATATTATATGTAATATAGTATATATTATGTAATATATTATATACTATATGA 5100
chimpanzee          ACATTATATATTATATATTATATATAACATATAATATATGTCATATAATATAATGTAT-- 1946
                    **** .*****::***** ::*****. *****:***.*.: ****:*****.*.***

human.10_000bp      TGTATATATTATATATTATAGAATGTATATTATATAATATGTTATAGAATGTATATATTA 5160
chimpanzee          -------ATTATATGTCATATTATATATATTAT--------TTATATAATTTAAATAATA 1991
                           *******.* *** :**.********        ***** *** **:***:**

human.10_000bp      TATAATATATAATGTATATATTATATAATGTATATATTATATAATGTATATATTATATAA 5220
chimpanzee          TATAATATATATAATATATAT---ATATTTTTAAAGGAAGAAAAATTTCAAAGGTACTC 2048
                    ***********::.*******   ***:* *:**:*  *:.:**:.*:*.:*: .** :.

human.10_000bp      TGTATAATGTATATATTATATAATATATTATATGTTGTATAATGTATATATTATATAATA 5280
chimpanzee          TGCTTGGT-ACAATAATCAAATATATAATTTGAGGCATAAAACATAACCATGAAACATAT 2107
                    ** :*..* :.:***:*.:*::*****:*:*.:*  .**:** .**:. ** *:* *:::

human.10_000bp      TATTATATG-TTATATAATGTATAATGTATATATTATATAATATATTATATGTTATATAA 5339
chimpanzee          TTATAACTGCATATGGAAAATACAGAGGATAATTTTTTCAATAACATATTTTTAAAACAT 2167
                    *::**:.** :***. **:.** *.:* ***::**:*: ****:.:***:* *:*:* *:

human.10_000bp      TGTATAATGTATATATTATATAATATATTATATGTTATATAATGTATAATGTATATATTA 5399
chimpanzee          TAACTAGTAATTTGGAAATATCGCATTTGGCAGGCCGGTATGAACATACCTTGAATGCAG 2227
                    *.:.**.*.::*: .::****.. **:* . * *  . :::.:. ***.  *.:**. :.

human.10_000bp      TATAATATATTATATATTACATATCCAATATATAATATATTATATATATCATATATCATA 5459
chimpanzee          CTACACAGGTTCCCCATATTATATTATATATATTATATATTATATGGATTATATGTTACA 2287
                     ::.* * .**. . **:: **** .:******:***********. ** ****.* * *

human.10_000bp      CATTATATATTATATATGATATATAACCTATAATATATAATATATATTATATATGTCATA 5519
chimpanzee          CAG---GTTCCCCATATGATATTATATGTAATATATTTTATATATATTAAATATG----- 2339
                    **    .*:  . *********:::*  **::****:*:*********:*****

human.10_000bp      TAGTATATATTATATGTCATATTATATATATTATATATTGTTCATATAACATATATTATA 5579
chimpanzee          TAATATATATTATATGTCATATTATATATTATATATGAT-----------ATATATTATA 2388
                    **.*************************::*****.:*           **********

human.10_000bp      TATGATGTATATTATATATTATATATAATATATTATGTATATAATATATTATATATTATA 5639
chimpanzee          TATGTCTTGTAT-ATATAATATATATTATATAATATATATTACATACACTATATGTAATA 2447
                    ****:  *.*** *****:*******:*****:***.***::.*** * *****.*:***

human.10_000bp      TATAATGTATATAATATGACGTATAATATATATAATGTATATAATTTGACATATAATATA 5699
chimpanzee          TATATTATAAATAATAC-----ATATTAAATATTATATATGATATATAATATATAATGTG 2502
                    ****:*.**:******      ***:**:****:**.***.::**:*.* *******.*.

human.10_000bp      TATAATGTATATAATTTGACATATAATATATATTATGTATATTATGTAATATATCATTAT 5759
chimpanzee          TATCATATATATTATATATATTATGTTTTATATATGATATATAATATATTATA-CAATAT 2561
                    ***.**.*****:**:*.:.:***.:*:*****:: .*****:**.**:**** **:***

human.10_000bp      ATTATATATATTATATATTATATATACATAATTTTTTTTTAGACAGAGTCCTGTTCTGTT 5819
chimpanzee          ATAATATATATTATATATTATGTATATATAATTTTTTTTTAGACAGAGTCTTGTTCTGTT 2621
                    **:*****************.**** ********************** *********

human.10_000bp      GCACAGGCTGGAATGCAATGGCGCCATCTTGGCTCACTGCAACCTCTGCCTCACGGGTTC 5879
chimpanzee          GCACAGGCTGGAGTGCAATGGCGCCATCTTGGCTCACTGCAATCTCTGCCTCACGGGTTT 2681
                    ************.*************************** ***************

human.10_000bp      AAGCGATTGTCGTGCCTCATCCTCCCTGGTCGCTGGGACTACACCACACTGGGACTACAC 5939
chimpanzee          AAGCGATTGTCCTGCCTCAGCCTCCCAGGTAGCTGGGACTACACCACACTGAGTCTACAC 2741
                    *********** ******* ******:***.******************.*:******

human.10_000bp      CAGCTGCCACCATGCCTGGCTAATTTTTCGTATTTTTAGTAGAGACAGGGTTTCACTGTA 5999
chimpanzee          CAGCTGCCACCATGCCTGGCTAATTTTTTGAATTTTTT-TAGAAATAGGATTTCACTGTA 2800
                    *************************** *:******: ****.* ***.**********

human.10_000bp      TTGGCCAGGATGGTCTTGATCTCCTCCCCTTTTGATCCTCTTGCCTTGGCCTCCCAAAGT 6059
```

```
chimpanzee          TTGGCCAGGATGGTCTTGATCTCCTCACCTTGTGATCCTCTTGCCTTGGCCTCCCAAAGT 2860
                    **************************.**** ****************************

human.10_000bp      GCTGGGATTACAGGCCTGAGCCAAGATACATATTTTTTAAAGGAAGAAAAATTTCAAAGG 6119
chimpanzee          GCTGGGATTACAGGCCTCAGCCAAGATACATATTTTTTAAAGGAAGAAAAATTTCAAAGG 2920
                    ***************** ******************************************

human.10_000bp      TACTCTGCTTGGTACAATAATCAAATATATAAATTGAGGAATAAAACATAACCATGAAAC 6179
chimpanzee          TACTCTGCTTGGTACAATAATCAAATATATAAATTGAGGAATAAAACATAACCATGAAAC 2980
                    ************************************************************

human.10_000bp      ATATTTATAACTGCCTATGGAAAATACAGAGGATAATTTTTTAAATAACATATTTTGAAA 6239
chimpanzee          ATGTTTATAACTGCATATGGAGAATACAGAGGATAATTTTTTAAAAAACGTATTTTGAAA 3040
                    **.***********.******.***********************:***.**********

human.10_000bp      GCATTAACTAGTAATTTGAAAAGATCGCATTTGGCAGGCCAGTATGAACATACCTTGAAT 6299
chimpanzee          GCATTAACTACCAATTTGAAAAGATCGCATTTGACAGGCCAGTATGAACATACCTTGAAT 3100
                    **********  ********************.***************************

human.10_000bp      GCAGCAAGACAGGTTCCCCATAAGAAAATTCAAAATCAGGGAATATGAAACCACAAAGGT 6359
chimpanzee          GCAGCTACACAGGTTCCCCATAAGAAAAATCAAAATCAGGGAAAATGAAACCACAAAG-- 3158
                    *****:* ********************:**************:**************

human.10_000bp      TCAATCTGCTCTGACCTTT-CAAAAACTCAGCACAGACAGTGGCACTTAGGACCAACGGC 6418
chimpanzee          -------GCTCTGACCTTTGTAAAAACTCAGCACAGACAGTGGCACTTAGGACCAACGGC 3211
                           ************  ***************************************

human.10_000bp      AGGAGATCCCTAATCCCATCACCATGGCGATAGGGCATAAACATTCCAGGGTGAAGACAC 6478
chimpanzee          AGGAGATCCCTAATCCCATCACCATGGCGATAGGGCATAAACATTCCAGGGTGAAGGCAC 3271
                    ********************************************************.***

human.10_000bp      AATCCACATTGGGAGGTCGAACTGCTGCCATGCAGACACGTGTGCTTTTCCATGTACAGG 6538
chimpanzee          AATCCACCTTGGGAGGTCCAACTGCTGCCATGCAGACAGGCGTGCTTTTACATGTACATG 3331
                    *******.********** ******************* * ********.******** *

human.10_000bp      AATGTCATTGAAGGCTCAGTGTTTTGTTTCAAAAACTGAATCCCAAGCTCACACATTATT 6598
chimpanzee          AAGGTCATTGAAGGCTCAGTGTTTTGTTTCAAAAACTGCTTCCCAAGCCGATACTTTATT 3391
                    ** ***********************************.:********  * **:*****

human.10_000bp      ATGCTGTGATTCTTGAAATAAGTTATGAGATGGGAAATAGGGCACCCCCAAATATAGCCA 6658
chimpanzee          ATGCTGTGCTGCTTAAAATAAGTTATGAGATGGGAAATAGGGCACCCCCAAATATAGCCA 3451
                    ********.* ***.*********************************************

human.10_000bp      ATAGTGAGAGTTTCAAATTGAAGAGAGGCACAACTGATACGTTGAGAATAAACAGAGATT 6718
chimpanzee          ATAGTGACAGTTTCAAATTGAAGAGAGGCACAACTGATACGTTGAGAATAAACAGAGATT 3511
                    ******* ****************************************************

human.10_000bp      CCATTCTGCTTTTTCTTTTTTAACTTTTATGTTAGATTCAGGGTGTACATGTGCAGG-TT 6777
chimpanzee          CCATTCTGGTTTTTCTTTTTCAACTTTTATGTTAGATTCAGGGTGTACATGTGCAGGTTT 3571
                    ******** *********** ************************************ **

human.10_000bp      TTTTCCTGGGTATATTGTGTGGTGCTGAGGTTTTGGGTGTGAATGATCCCAACACCCAGG 6837
chimpanzee          TTTACCTGGGTATATTGTGTGGTGCTGAGGTTTGAGGTGTGAATGATCCCAACACCCAGG 3631
                    ***:***************************** .*************************

human.10_000bp      TACTGAACATGGTACTCAGCAGTTTTTCAACCTTTTCCTTCCTCCCTCCCCCTCCTAGCA 6897
chimpanzee          TACTGAACCTGGTACTCAGCAGTTTTTCAACCTTTTCCTTCCTCCCTCCCCCTCCTAGCA 3691
                    ********.***************************************************

human.10_000bp      G-TCCTAGTGTCTATTGTCACCATCTTCATGTCCATGGGTACTCAGAATTTAGCTCCTAC 6956
chimpanzee          GTCCCTAGTGTCTGTTGTCACCATCTTCATGTCCATGGGTACTCAGAATTTAGCTCCTAC 3751
                    *  **********.**********************************************

human.10_000bp      TTATAGGAACATGAGGCGTTTGTTTTCTTTTACTGCATTAGTTCACTTCGTGGATTCCAG 7016
chimpanzee          TTACAGGAACATGAAGCGTTTGTTTTCTTTTACTGCATTAGTTCACTTCGTGGATTCCAG 3811
                    *** **********.*********************************************

human.10_000bp      CTCTAGACATTTTCCCTCAAAGAACATAATTTCATTCGTTTTTGTGGCTGCATAGTATTC 7076
chimpanzee          CTCTAGACATTTTCCCTCAAAGAACATAATTTCATTCTTTTTTGTGGCTGCATAGTATTC 3871
                    ************************************* **********************

human.10_000bp      CATGGTCTATATGTACCACATTTTTATCCAGTCCACTGTTGATGGGCACCTAGGTTGATC 7136
chimpanzee          CATGGTCTATATGTACCACATTTTTATCCAGTCCACTGTTGATGGGCACCTAGGTTGATC 3931
                    ************************************************************

human.10_000bp      CCATGTCTTTGCTAATGTGAATAGTGTTGCAATAAACATATAAGCGCATACGTCTTTTTG 7196
chimpanzee          CCATGTCTTTGCTAATGTGAATAGTGTTGCAATAAACATATAAGCGCATACGTCTTTTTG 3991
                    ************************************************************

human.10_000bp      GTGGAATGATTTGTTTTCTTTTGGATACATATTCAGTAATGAGACTGCTGGGTTGAATGT 7256
chimpanzee          GTGGAATGATTTGTTTTCTTTTGGATACATATTCAGTAATGAGACTGCTGGGTTGAATGT 4051
```

```
                    ************************************************************

human.10_000bp      TAGTTCTGTTTTATGTTCTTTGAGAAATCTCCAAATTGCTTTCCACAGTGGCTGAACTAA 7316
chimpanzee          TAGTTCTGTTTTATGTTCTTTGAGAAATCTCCAAATTGCTTCCCACAGTGGCTGAACTAA 4111
                    ***************************************** ******************

human.10_000bp      CTTACATCCCCACCAAAGGTGTATAAGCATTCCCTTTTCTCCTTATCCTTGCCAGTATCT 7376
chimpanzee          CTTACATTCCCACCAAAGGTGTATAAGCATTCCCTTTTCTCCTTATCCTTGCCAGTATCT 4171
                    ******* ****************************************************

human.10_000bp      GCTATTTTTTTTTTTTT----ACTTTTTAAAAATAGCCATTCTGACTAGTGAGAGATAAT 7432
chimpanzee          GCTATTTTTTTTTTTTTTTTGACTTTTTAAAAATAGCCATTCTGACTGGTGAGAGATAAT 4231
                    *****************    **************************.************

human.10_000bp      ATCTCATTGTGGTTTTGATTTGCATTTCTCTCGTGATTAGTGATGATGAGCATTTTTTTC 7492
chimpanzee          ATCTCATTGTGGTTTTGATTTGCATTTCTCTCGTGATTAGTGATGATGAGCATTTTTTTC 4291
                    ************************************************************

human.10_000bp      ATGTTTGTTGGCTGCATGTGTGTCTCCTTCTGAGAAGTGTCTATGTCTTTTCCCCCTTTT 7552
chimpanzee          ATGTTTGTTGGCTGCATGTGTGTCTCCTTCTGAGAAGTGTCTGTGTCTTTTGCCCCTTTT 4351
                    ******************************************.******** ********

human.10_000bp      TAAATGGGGTTGTGTTTTGCTTGTTGAATTATGTTCCTTATAGATTCTAAATATTAGACC 7612
chimpanzee          TAAATGGGGTTGTGTTTTGCTTGTTGAATTATGTTCCTTATAGATTCTAAATATCAGACC 4411
                    ****************************************************** *****

human.10_000bp      TTTGTTGGATGCATAGTTTGTGAATAATTTCCCCCATTCTGTAGGTTGTTTACTCTGCTG 7672
chimpanzee          TTTGTTGGATGCATAGTTTGTGAATAATTTCCCCCATTCTGTAGGTTGTTTACTCTGCTG 4471
                    ************************************************************

human.10_000bp      ATGGTTTCCTTTGCTGTGTGGCAGCTCTTTAGTTTAATTAGGTCCCATTTGTCAACTTTT 7732
chimpanzee          ATGGTTTCCTTTGCTGTGTGGCAGCTCTTTAGTTTAATTAGGTCCCATTTGTCAACTTTT 4531
                    ************************************************************

human.10_000bp      GCTTTTGTTGCAATTTCTTTTGAAGACTTAGTCATGAATTATTTCCCATAGCCCATATCC 7792
chimpanzee          GCTTTCGTTGCAATTTCTTTTGAAGACTTAGTCATGAATTATTTCCCATAGCCCATATCC 4591
                    ***** ******************************************************

human.10_000bp      AGAATGGTACTTTTGAAGTTTTTCTTCTAGGATTATTGTAGTTTGAGGTCTTAAATTTAA 7852
chimpanzee          AGAATGGTACTTTTGAAGTTTTTCTTCTAGGATTATTGTAGTTTGAGGTCTTAAATTTAA 4651
                    ************************************************************

human.10_000bp      ATCTTTAATCCAACCTGAGTTAATTTTTGTATATGGTGAAAAGGTGACCAGTTTTTTGTT 7912
chimpanzee          ATCTTTAATCCAACCTGAGTTAATTTTTGTATATGGTGAAAAGGTGACCAGCTTTTTGTT 4711
                    *************************************************** ********

human.10_000bp      TTTTGTTTTTGTTTTTTGTTTGTTTGTTTGTTTTTTGAGAGGTAGTCTTCCTCTGTCACC 7972
chimpanzee          TTTTGTTTTTGTTTTTTGTTTGTTTGTTTGTTTTTTGAGAGGTAGTCTCCCTCTGTCACC 4771
                    ************************************************ ***********

human.10_000bp      AGGCTGTAGCAGAGTGGCGCCATCTCAGCTCACAGCAACCTCCACCTGCCGGGTTCAAGC 8032
chimpanzee          AGGCTGTAGCAGAGTGGCGCCATCTCAGCTCACAGCAACCTCCACCTGCCGGGTTCAAGC 4831
                    ************************************************************

human.10_000bp      GCTTCTCCTGCCTCAGCTTCCTGAGTCGCTGGGATTACAGGCACGTGCCACCACACCCAG 8092
chimpanzee          GATTCTCCTGCCTCAGCTTCCTGAGTCACTGGGATTACAGGCACGTGCCACCACACCCAG 4891
                    *.*************************.********************************

human.10_000bp      CTAATTTTTGTATTTTTAGTAGAGACAGGGTTTCACCATGTTGGCCGGGCTGGACTTGAT 8152
chimpanzee          CTAATTTTTGTATTTTTAGTAGAGACAGGGTTTCACCATGTTGGCCGGGCTGGACTCGAT 4951
                    ******************************************************** ***

human.10_000bp      CTTCTGACCTTGTGATCCTCCCACCTTGGCCTCCCAAAGTGCTGGGATCACAGTCGTGAG 8212
chimpanzee          CTTCTGACCTTGTGATCCTCCCACCTTGGCCTCCCAAAGTGCTGGGATCACAGTCGTGAG 5011
                    ************************************************************

human.10_000bp      CCACTGTGCCTGGCCAATAAGGTGCATTATTAACATCAATAAAGCTCAGGAAACAGCTTT 8272
chimpanzee          CCACTGTGCCTGGCCAATAAGGTGCATTATTAACATCAATAAAGCTCAGGAAACAGCTTT 5071
                    ************************************************************

human.10_000bp      CACCATATTTTTGTTTAATTTACAGTTTTTCCCAGAGTCTTTGGAATAGATTCCTCCCTC 8332
chimpanzee          CACCATATTTTTGTTTAATTTACAGTTTT---------CTTTGGAATAGATTCCTCCCTC 5122
                    *****************************         **********************

human.10_000bp      CATGAGCCAGAGAACTTACAATGTTCACTACAGTGTCTTTAAATGTAGCAGTAGCAGCTG 8392
chimpanzee          CATGAGCCAGAGAACTTACAATGTTCACTGCAGTGTCTTTAAATGTAGCAGTAGCAGCTG 5182
                    *****************************.******************************

human.10_000bp      TGGGTTGAGAACACAAGTCTTCGACTCTTCCTTTTAGAGCCAGCCATTCACGGTGCTCTG 8452
chimpanzee          TGGGTTGAGAACACAAGTCTTCGACTCTTCCTTTTAGAGCCAGCCATTCAAGGTGCTCTA 5242
                    **************************************************.********.
```

```
human.10_000bp      TGTTCTTATTCGGCATTGGGTAGGGGCATCTGGGTGCTGGGCATAGCGCCAGTGCCCCAT 8512
chimpanzee          TGTTCTTATTCGGCATTAGGTAGGGGCATCTGGGTGCTGGGCATAGCACCAGTGCCCCAT 5302
                    *****************.*****************************.************

human.10_000bp      GGAGGAGGGAGGCAGGAAGCCCCTTTCTTCCTTCTCTAAAACCTTTTTCTTTATGTAGA 8572
chimpanzee          GGAGGAGGGAGGCAGGAAGCCCCTTTCTTCCTTCTCTAAAACCTTTTTCTTTATGTAGA 5362
                    ************************************************************

human.10_000bp      TCCAAGTTTTTAGCCTATATCATTTTCTTCTCTCTGGATAACTTCTTTTAACATTTTGAC 8632
chimpanzee          TCCAAGTTTTTAGCCTATATCATTTTCTTCTCTCTGGATAATTTCTTTTAACATTTTGAC 5422
                    ***************************************** ******************

human.10_000bp      AGATCTACTGACAGCAAAATTGCATTTCTCTGAGAAAGTCTTTATTTTTCTTCACTTTGG 8692
chimpanzee          AGATCTACTGACAGCAAAATTGCGTTTCTCTGAGAAAG----TATTTTTCTTCACTTTGG 5478
                    ***********************.**************    ******************

human.10_000bp      AAAGATAATTTTGCAGGATACAGAATTTTAGGTTGGTGGAATTTTCTTGCTTGCTTGCAT 8752
chimpanzee          AAAGATAATTTTGCAGGATACAGAATTTTAGGTTGGTGGAATTTTCTTGCTTGCTTGCAT 5538
                    ************************************************************

human.10_000bp      GGGTTCTGAAGAAAAGTTTGATGCAATTTTTATTCTTATCCTTACATGTGTTAGGCCCTG 8812
chimpanzee          GGGTTCTGAAGAAAAGTTTGATGCAATTTTTATTCTTATCCTTACATGTGTTAGGCCCTG 5598
                    ************************************************************

human.10_000bp      CTAAAGCCCAAGGTGGTTAGACTCTTGTGAAATAGTTTCCCTGGGGCAGGCTTTTGCTAA 8872
chimpanzee          CTAAAGCCCAAGGTGGTTAGACTCTTGTGAAATAGTTTCCCTGGGGCAGGCTTTTGCTAA 5658
                    ************************************************************

human.10_000bp      GGG-AACGGAACACCCAGGGTGTATTTCAAAGTAGTTACTTTTCCCCTTCCCTGTGGGAA 8931
chimpanzee          GGAGAACGGAACACCCAGGGTGTATTTCAAAGTAGTTACTTTTCCCCTTCCCTGTGGGAA 5718
                    **. ********************************************************

human.10_000bp      GCAGGAGGGAATTTTTCTCTGATGTGCATAAGAGCGTCTGGCAGAGCTCTTGGAGGTTCA 8991
chimpanzee          GCAGGAGGGAATTTTTCTCTGATGTGCATAAGAGCGTCTGGCAGAGCTCTTGGAGGTTCA 5778
                    ************************************************************

human.10_000bp      TGAAAGTGTAAGGGCCCCCTAAGACCGGGCTCCCTTAATTCTTAATTCTCAAGTTTATGT 9051
chimpanzee          TGAAAGTGTAGGG-CCCCCTAAGACCGGGCTCCCTTAATTCTTAATTCTCAAGTTTATGT 5837
                    **********.** **********************************************

human.10_000bp      ACACGTGGCCTCCAGAAATTTGTTGTTTACATGTAAGTCTTCCCACCGTGGTTCTGGCTC 9111
chimpanzee          GCATGGAGCCTCCAGCGATTTGTTGTTTACATGTAAGTCTTCCCACCGTGGTTCTGGCTC 5897
                    .** * .********..*******************************************

human.10_000bp      CAGTGG-CAGCCTCCGATCCTGTTAGGCTGGGACTCACTGCCTCTCCAATTTGGGGGATA 9170
chimpanzee          CAGTGGCCAGCCTCCGATCCTGTTAGGCTGGGACTCACTGCCTCTCCAATTTGGGGGATA 5957
                    ****** *****************************************************

human.10_000bp      ATGGTTTGGCGTGTTAACCTCAAGTCTCTGATGGATCTAAGAAAAGTTGTTAATTTTCAG 9230
chimpanzee          ATGGTTTGGCGTGTTAACCTCAAGTCTCTGATGGATCTAAGAAAAGTTGTTAATTTTCAG 6017
                    ************************************************************

human.10_000bp      TTTGTTCAAGGTGTTTTTTGTTGTTGTTGTGAGGACAGGAGTGACAGCTTCCAAGCTGGA 9290
chimpanzee          TTTGTTCAAGGTGTTTTTTGTTGTTGTTGTGAGGACAGGAGTGACAGCTTCCAAGCTGGA 6077
                    ************************************************************

human.10_000bp      AAACAGAAGTCACATTTGGGTTTTGTTTCAAGATATTTTTCCAATGACAGAGAATGATGC 9350
chimpanzee          AAACAGAAGTCACATTTGGGTTTTGTTTCAAGATATTTTTCCAATGACAGAGAATGATGC 6137
                    ************************************************************

human.10_000bp      CACACACATTTCCATAGATGTCTTTTGATGCAGACGTGCATGTGTTTCTGTTATCCTAAG 9410
chimpanzee          CACACACATTTCCATAGATGTCTTTTGATGCAGACGTGCATGTGTTTCTGTTATCCTAAG 6197
                    ************************************************************

human.10_000bp      AGTGAAATACCTGAGTCCTGAATGTGCTCCTTTCTAGAACCTTTATTCAATGCCAAACAC 9470
chimpanzee          AGTGAAATACCTGAGTCCTAAATGTGCTCCTTTCTAGAACCTTTATTCAATGCCAAAAAC 6257
                    ******************.**************************************.**

human.10_000bp      AGTATCCCCTCCAGCAGTGATGAGAGTTCTTGTTGCTCCAGATTCTCTCCAACACTTGGT 9530
chimpanzee          AGTATCCCCTCCAGCAGTGATGAGAGTTCTTGTTGCTCCAGATTCTCTCCAACACTTGGT 6317
                    ************************************************************

human.10_000bp      GTTCTGTCTTTTACATTTTAGCCGTTTTGGAGGATATGTTATCCTGTTGTGATGTTAATT 9590
chimpanzee          GTTCTGTCTTTTACATTTTAGCCGTTTTGGAGGATATGTTATCCTGTTGTGATGTTAATT 6377
                    ************************************************************

human.10_000bp      TGCATTTACATGAATATTAAGAAAGCTGAACACATTCAAGTATTTGCTTTTATAAATTAC 9650
chimpanzee          TGCATTTACATGAATATTAAGAAAGCTGAACACATTCAAG-TTTTGCTTTTATAAATTAC 6436
                    **************************************** :******************
```

```
human.10_000bp      ATGTTTAAG-TGTTTTACCCATTGTTCTTTCTCTTAGTGATTTTTTTTTTTTTCCCAAGGC 9709
chimpanzee          ATGTTTAAAGTGTTTTACCCATTGTTCTTTCTCTTAGTGATTTTT---------AAGAGC 6487
                    ********. ***********************************         .*..**

human.10_000bp      AGAAGAATTTTTCTTAGTACAGAACAAAATGAAAAGTCTCCCATGTCTACTTCTTTCTAC 9769
chimpanzee          TCTTTACATATTGTTG-ATGTGAGCCCTCTGTCAG---TTCTATGTGTTCATAGATCTGT 6543
                    : :: *.:*:** **. :: :**.*..:.**:.*.   * * **** *:*:*. :***. 

human.10_000bp      ACAGACACGGCAACCATCCGATTTCTCAATCTTT-TCCCCACCTTTCCCCCCTTTCTATT 9828
chimpanzee          TCCCACTCTGCAACTTGCC-TTGTCACTTTCTTAGTCTCTTAATAAACAGAAATTCTTAA 6602
                    :*. **:* ***** : ** :* **:*::****: ** * :..*::.*. ..:****:::

human.10_000bp      CCACAAAACCGCCATTGTCATCATGG----CCC-----------GTTCTCAATGAGCTGT 9873
chimpanzee          TTTGAATACAGTCTAATTTATCAATTTATTCCCTTAAAACTATGGCTCTTTGTGAACTGT 6662
                      : **:**.* *::: * ****:      ***           * *** :.***.****

human.10_000bp      TGGGTACACCT---CCCAGACGGGGTGG-------TGGCCGGGCAGAGGGGCTCCTCACT 9923
chimpanzee          TTAAGAAATCTGTCCCCATTCATGGTCATAAAAATTGGCTGGGCATGGTGGCTCACACCT 6722
                    * .. *.* **   **** :*. *** .       **** ***** .* *****. ..**

human.10_000bp      ----TCCCAGTAGGGGCGGCCGGGCAGAGGCGCCCCTCACCTCCCGGACGGGGCGGCTGG 9979
chimpanzee          GTAATCCCAGCACTTTGGGAGATCAAGGTGGGCAGATCACCTG-AGATCAGGAGTTCGAG 6781
                        ****** *     **. .  .**. * **. .******  .*.:*.**.   * .*

human.10_000bp      CCGGGCTGGGGGCTGACCCCC 10000
chimpanzee          ACCAGCTG-------------- 6789
                    .* .****
```
