# Supplementary figures and images for: Complexity of a small non-protein coding sequence in chromosomal region 22q11.2: presence of specialized DNA secondary structures and RNA exon/intron motifs
Source: BMC Genomics. 2015 Oct 14;16:785. doi: 10.1186/s12864-015-1958-6 (PMC4607176; doi:10.1186/s12864-015-1958-6)

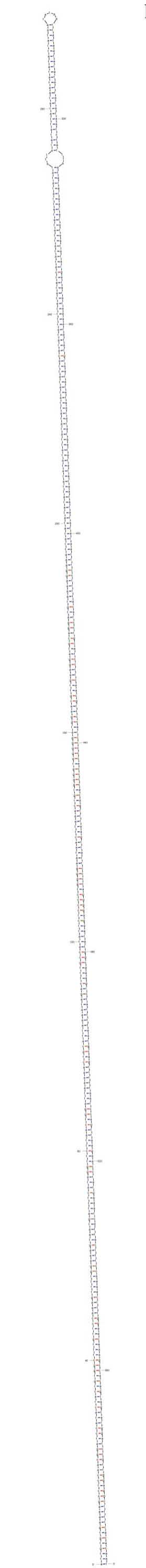

Supplement: Additional file 2: Figure S2. — Secondary structure that yields high frequency of translocation. Nucleotide sequence is from PATRR Type C, accession AB 538237. Secondary structure determined by DNA mfold program [36]. (JPEG 48 kb) [file 12864_2015_1958_MOESM2_ESM.jpeg]

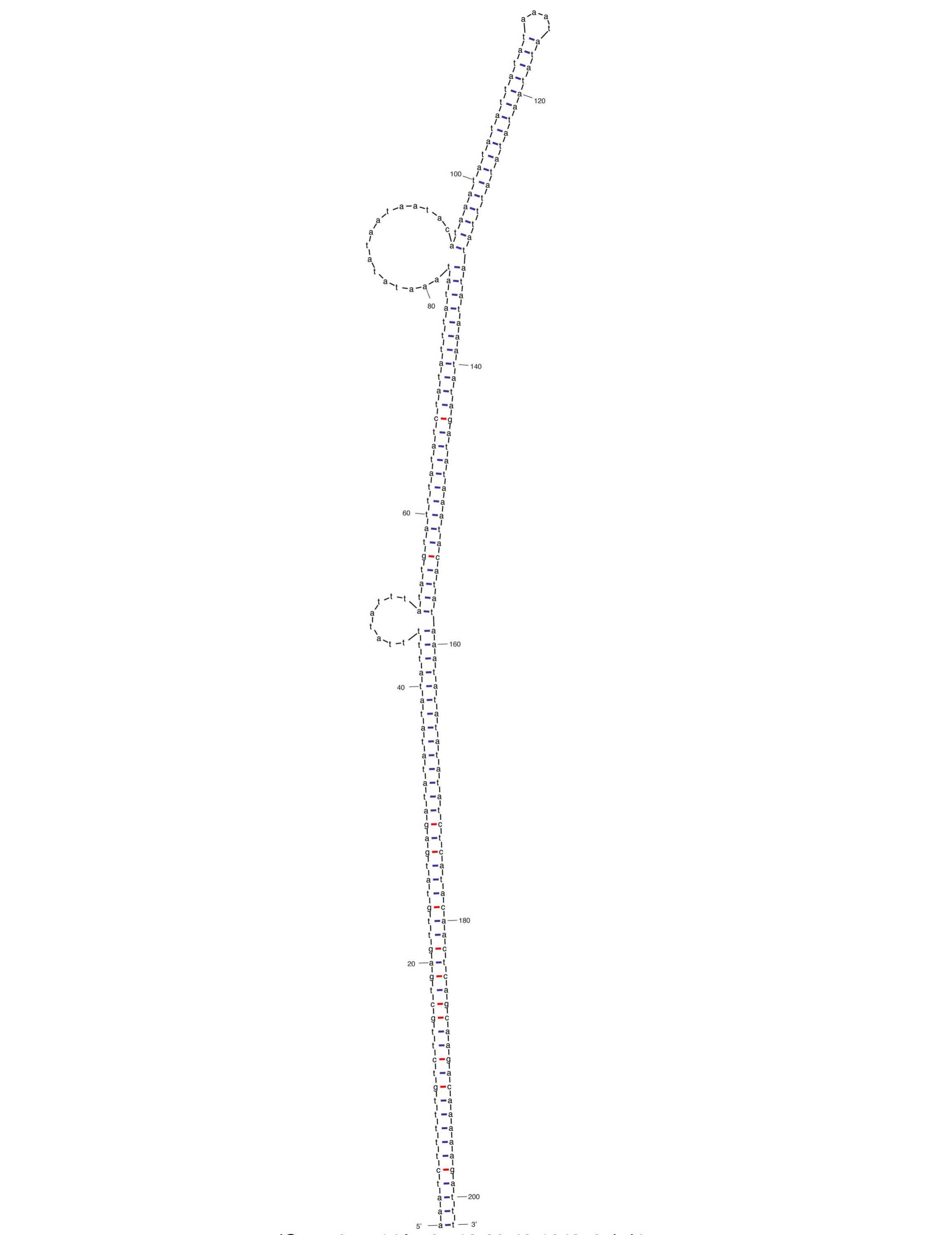

Supplement: Additional file 3: Figure S3. — Example of a secondary structure that yields very low frequency of translocation. Sequence is from accession AF 391128 on chromosome 11. Secondary structure determined by DNA mfold program [36]. (JPEG 164 kb) [file 12864_2015_1958_MOESM3_ESM.jpeg]

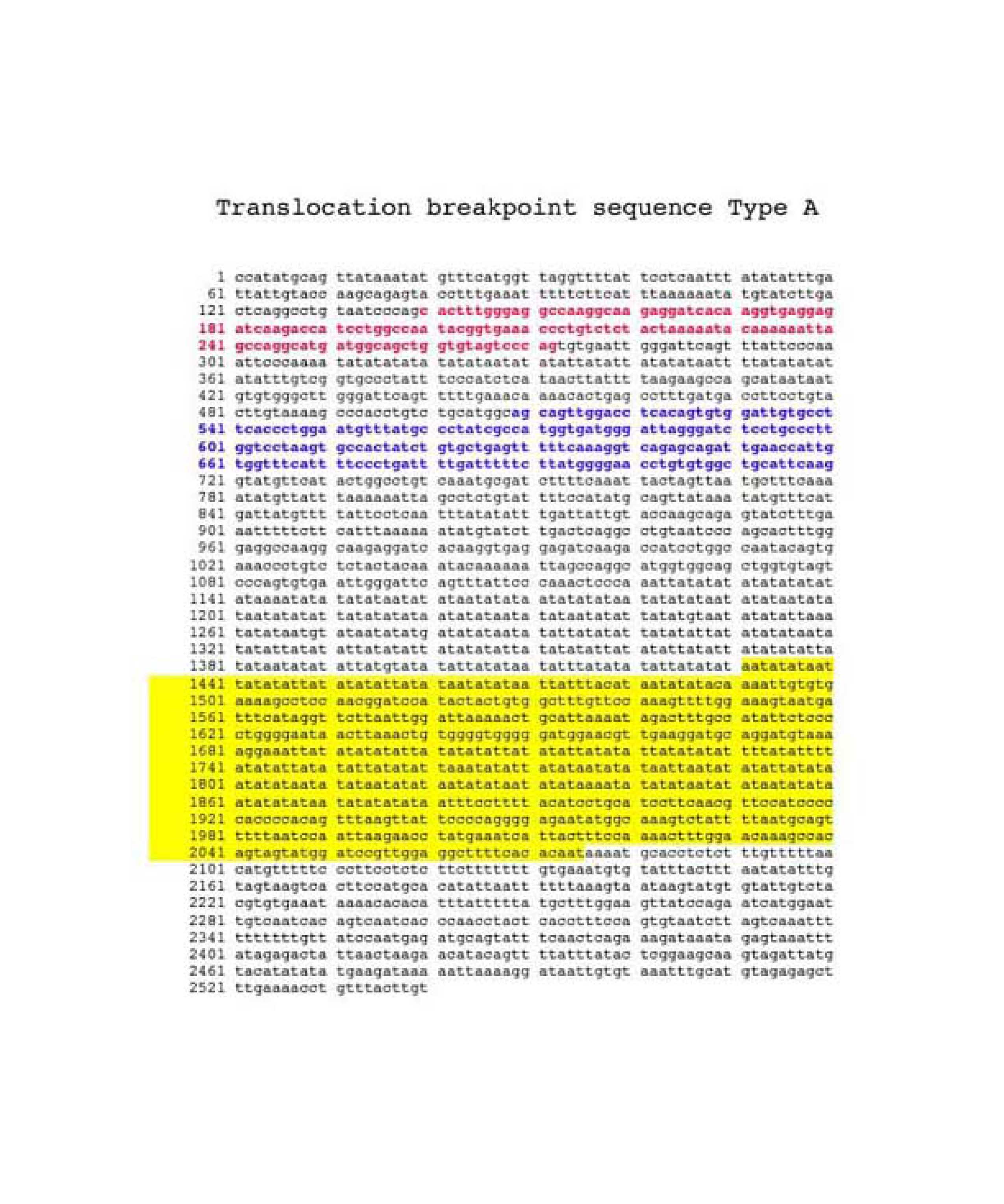

Supplement: Additional file 4: Figure S4. — Translocation breakpoint and flanking sequences, Type A from Accession GenBank: AB261997.1. Color code: red, intron sequence; blue, exon 1 sequence; yellow, translocation breakpoint hot spot stem loop sequence (PATRR). (JPEG 502 kb) [file 12864_2015_1958_MOESM4_ESM.jpeg]

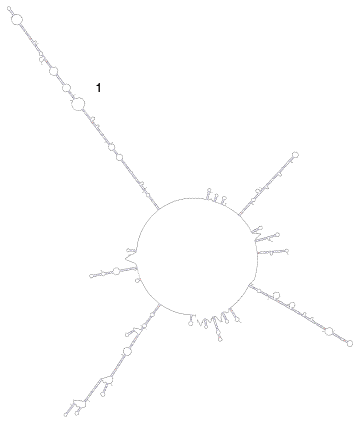

Supplement: Additional file 5: Table S1. — Folded random sequences- stem bp, lengths. (TIFF 303 kb) [file 12864_2015_1958_MOESM5_ESM.tiff]
