# Supplementary material for: Complexity of a small non-protein coding sequence in chromosomal region 22q11.2: presence of specialized DNA secondary structures and RNA exon/intron motifs
Source: BMC Genomics. 2015 Oct 14;16:785. doi: 10.1186/s12864-015-1958-6 (PMC4607176; doi:10.1186/s12864-015-1958-6)
Supplement: Additional file 6: Figure S5. — Longest and best formed stem loop (stem loop 1) from a sample of 40 random sequences. Random sequence of 1393 nt, 8.3 % G + C. Sequence folded by mfold [36]. (DOCX 53 kb) [file 12864_2015_1958_MOESM6_ESM.docx]

**Table S1. Folded random sequences-stem bp lengths***

| Random trial number** Number of bp Number of protrusions in helix*** |
| --- |
|  |
| 1 74 6 |
|  |
| 2 75 8 |
|  |
| 3 85 8 |
|  |
| 4 123 14 |
|  |
| 5 74 7 |
|  |
| 6 72 6 |
|  |
| 7 77 11 |

* only stems >70 bp shown

**total of 40 random trials

***looped-out positions, bulged positions, and protruding mini-stem-loops
